# Environmental Acceptability of Geotechnical Composites from Recycled Materials: Comparative Study of Laboratory and Field Investigations

**DOI:** 10.3390/ijerph20032014

**Published:** 2023-01-21

**Authors:** Marija Đurić, Vesna Zalar Serjun, Ana Mladenovič, Alenka Mauko Pranjić, Radmila Milačič, Janez Ščančar, Janko Urbanc, Nina Mali, Alenka Pavlin, Janez Turk, Primož Oprčkal

**Affiliations:** 1 Slovenian National Building and Civil Engineering Institute, Dimičeva ulica 12, 1000 Ljubljana, Slovenia; 2 Jožef Stefan International Postgraduate School, Jamova 39, 1000 Ljubljana, Slovenia; 3 Department of Environmental Sciences, Jožef Stefan Institute, Jamova 39, 1000 Ljubljana, Slovenia; 4 Geological Survey of Slovenia, Dimičeva ulica 14, 1000 Ljubljana, Slovenia; 5 Termit d.d., Drtija 51, 1251 Moravče, Slovenia

**Keywords:** waste, recycling, lysimeter, potentially hazardous substances, immobilization, revitalisation

## Abstract

The environmental properties of three geotechnical composites made by recycling wastes were investigated on a laboratory scale and in the field with the use of lysimeters designated for the revitalization of degraded mining sites. Composites were prepared by combining the mine waste with paper-mill sludge and foundry sand (Composite 1), with digestate from municipal waste and paper ash (Composite 2), and with coal ash, foundry slag and waste incineration bottom ash (Composite 3). The results of laboratory leaching tests proved that Composites 1 and 3 are environmentally acceptable, according to the legislative limits, as the potentially hazardous substances were immobilized, while in Composite 2, the legislative limits were exceeded. In the field lysimeters, the lowest rate of leaching was determined for optimally compacted Composites 1 and 3, while for Composite 2 the leaching of Cu was high. This study proved that optimally installed Composites 1 and 3 are environmentally acceptable for use in construction as an alternative to virgin materials, for the revitalization of degraded mining sites or, along with Composite 2, for closure operations with landfills. In this way, locally available waste streams are valorised and channelized into a beneficial and sustainable recycling practice.

## 1. Introduction

Although waste prevention has been set as a priority in the EU’s waste-management hierarchy [1], the generation of waste cannot be avoided in many human activities. Fortunately, different forms of waste could be used as secondary resources. This is in line with the concepts of sustainable development and the circular economy that are embedded in the long-term strategy of the EU [2]. Establishing a circular economy based on the responsible use of materials, water, and energy was recognized as an opportunity for resource-insufficient Europe to maintain access to vital resources, maintain its global competitiveness, and ensure a high-quality environment [3]. However, the quantity of materials from waste steams is not sufficient to support a circular economy [4] and the mining of natural resources is still of vital importance, even though it has many adverse impacts on the environment, causing deforestation, erosion, contamination and the alteration of soil, local water bodies and wetlands, dust emissions, and land degradation [5]. Consequently, mining activities have a negative social perception, especially in the case of open-pit mining, which results in up to ten times more land degradation than underground mining [6].

Reclamation activities are emphatically enforced by the regulations in most countries. According to EU law [7], Member States are obliged to demand that mining operators include site-rehabilitation measures in their projects. The most frequent approach to the rehabilitation of open pits is backfilling with extractive waste or the installation of construction products made from recycled waste in earth structures in order to restore the original morphology or to establish areas for urbanization [8]. In the latter case, the waste to be recycled is often combined with other waste or natural materials to produce geotechnical composites with suitable mechano-physical characteristics and to reduce the level of potentially hazardous substances (PHSs) below the legislative limits, all in order to comply with the legal, environmental, and technical demands [9,10,11].

In many Member States, the environmental acceptability of recycled waste-based construction products is determined based on laboratory leaching procedures, which gives information about whether the material is compliant with the arbitrary legislative limits. These standardised tests might not reflect exactly the behaviour of the investigated material in natural conditions [12]. Field experiments using lysimeters are designed to provide the most straightforward information about the potential environmental impacts of investigated materials in the natural environment by monitoring the fate and mobility of PHSs, depending on the geographical weather conditions and the quality of the installation. At present, there is no legislation that would regulate the environmental acceptability of materials with the use of such tests, which are currently used mostly for risk-assessment studies. [12,13].

Due to the general increase in the volume of waste production, attention is paid to the issues of waste management. The circular economy and resource efficiency imply the minimization of material losses and the maximization of material circulation. When waste generation cannot be prevented, it should be used as a resource [14]. Based on a review done by Mohammad A. Al-Ghout et. all [15], in terms of the technical advantages and disadvantages, the incineration of municipal solid waste (MSW) appears to be the best choice compared to other management solutions (e.g., digestion, composting, landfilling). Nevertheless, landfilling is still one of the most common methods of municipal waste management. As for the incineration procedure, dealing with incinerated municipal waste, i.e., bottom and fly ash, has become one of the biggest challenges. The physical and chemical characteristics of these ashes are not easy to generalize, and their uses are different. With a different pre-treatment, ashes can be used as alternatives for the production of lightweight aggregate in the construction field [16] or as recycled aggregate in road construction [17]. The use of incinerated MSW (bottom and fly ash) for the preparation of geopolymers has also been reported in recent years [18,19,20]. For industrial waste recycling, treatment, incineration, and landfill are the current practices used in waste management. Recycling and treatment of solid or liquid industrial wastes usually produces non-hazardous residues and they are generally used on site. Industrial waste is also used, as is incinerated MSW, in the civil construction sector. [21]

Three construction products, referred to here as geotechnical composites, were developed through the use of recycled waste materials. Industrial and municipal wastes, i.e., paper-mill sludge and paper ash, coal ash, solid-waste incineration bottom ash, foundry slag and sand, digestate from the mechanical and biological treatment of municipal waste, were used in combination with Termit’s mine waste. They were designated for the rehabilitation of abandoned open-pit mining sites, for the company Termit d.d., which is one of the largest Slovenian mining companies dealing with the extraction and processing of silica sand and the production of auxiliary casting materials for foundries and ironworks.

The aim of this study was to investigate the environmental acceptability of three construction products based on recycled waste for final use as geotechnical composites, designated for the rehabilitation of abandoned open-pit mining sites at Termit d.d. Both laboratory and field investigations large enough to reliably reflect the conditions of earth structures on a large scale were made using lysimeters. Percolated water from the lysimeters was periodically collected over one year and analysed to evaluate the release of PHSs into the environment. The experimental field data were supported and compared with the data from the laboratory leaching tests.

The optimal installation of the geotechnical composites made from recycled materials is of vital importance to ensure their environmental acceptability [22]. Therefore, the influence of installation, i.e., the compaction efficiency, on the release of PHSs from the composites was also investigated.

## 2. Materials and Methods

### 2.1. Raw Materials

Selected non-hazardous wastes were used as raw materials for the experiments in this study:

Paper-mill sludge (PMS) is composed of 95% dewatered residues, made up of rejected cellulose fibres and mineral fillers from the chemical–mechanical processes in paper production, and 5% dewatered sludge from a biological treatment plant for purification of industrial wastewater from the paper mill.

Paper ash (PA) was also obtained from the paper mill, where it was formed in a steam boiler, in which the de-inking sludge from the paper recycling process and the sludge from the treatment of paper-mill wastewater were incinerated. This ash was a mixture of 90% bottom ash and 10% fly ash.

Coal fly ash (CA) was formed in a heating plant during co-combustion of coal (76%), biomass (21%) and paper sludge (up to 3%). The CA sample was collected in filters for air-pollution control.

Solid-waste incineration bottom ash (SWIA) is a coarse-grained material that consist of larger particles of burnt and charred waste metals and glass. It was formed in an incinerator for industrial and mixed-municipal solid wastes.

Foundry slag (FSL) was obtained from cast-iron production after being formed in a cupola furnace. The FSL was a coarse-grained material with some grains up to 100 mm in size.

Foundry sand (FS) was obtained from disintegrated moulds and cores used in the production of ferrous metal castings. The moulds and cores consisted of quartz sand with organic and inorganic binders.

Digestate (DI) from mechanical-biological anaerobic treatment of mixed municipal solid waste was used. The digestate sample was a coarse-grained material, consisting of a heterogeneous mixture of organic (29%) and inorganic (71%) components.

Mine waste (MW) was generated during the processing of quartz sand at Termit. It was clayey sandy silt that was separated from the quartz sand in a washing process and later dewatered in a filter press.

Detailed information about the source and EWL (European Union’s List of Wastes) classification of the raw materials is given in Table S1.

A flow chart of the experimental set-up is given in Figure S1.

### 2.2. Geotechnical Composites

Three geotechnical composites were prepared by mixing the raw materials in ratios based on the dry-mass content, as presented in Table 1.

The composition of the geotechnical composites was carefully tailored by taking into account the waste properties, the technical requirements for geotechnical reclamation fill, as well as the type and quantities of waste for which Termit has permission to collect and recycle.

#### 2.2.1. Preparation of Laboratory Samples

First, the maximum reference dry density and the optimal water content in the composites according to the Proctor compaction test were determined [23]. These are the basic geotechnical parameters required to ensure the optimal installation of the composites by compaction, so that the mechanical stability and immobilization of the potentially toxic substances (PTS) are achieved. The maximum dry density and optimal water content, i.e., a gravimetric water content, which is the ratio between the masses of water and dry matter in composite, are given in Table S2 for each composite.

**Figure 1:**
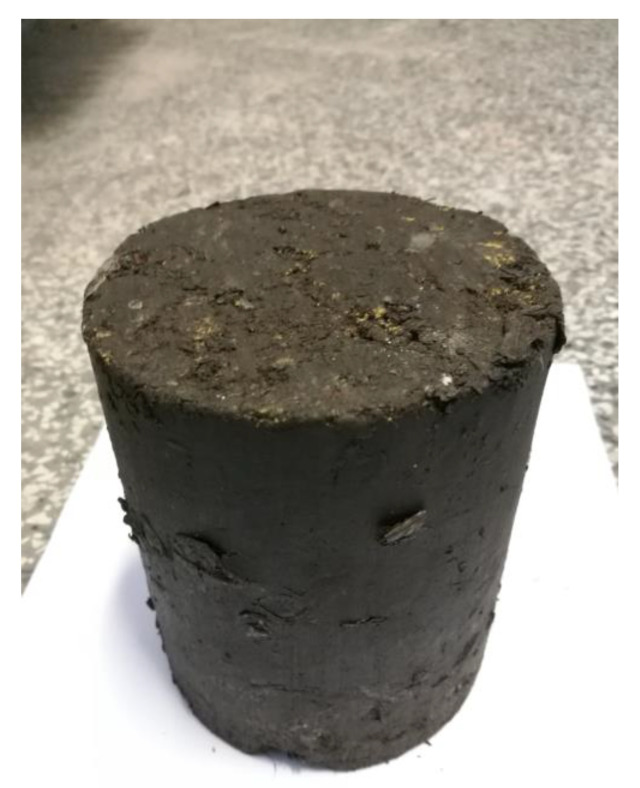
An example of laboratory sample of the geotechnical Composite 2.

To conduct a laboratory investigation of the environmental acceptability of composites, all the composites were prepared in the laboratory in the form of monolithic cylindrical test samples of 10 cm in diameter and 12 cm in height (Figure 1).

The laboratory samples were then cured in a climatic chamber under constant temperature and humidity conditions (22 ± 2 °C and 98% humidity) for 28 days. After curing, the samples were subjected to a tank leaching test, according to procedure SIST EN 1744-3:2002 [24] (liquid-to-solid ratio (L/S) = 10:1). Demineralised water was used as the leaching agent.

#### 2.2.2. Field Installation of Geotechnical Composites into Lysimeters

**Figure 2:**
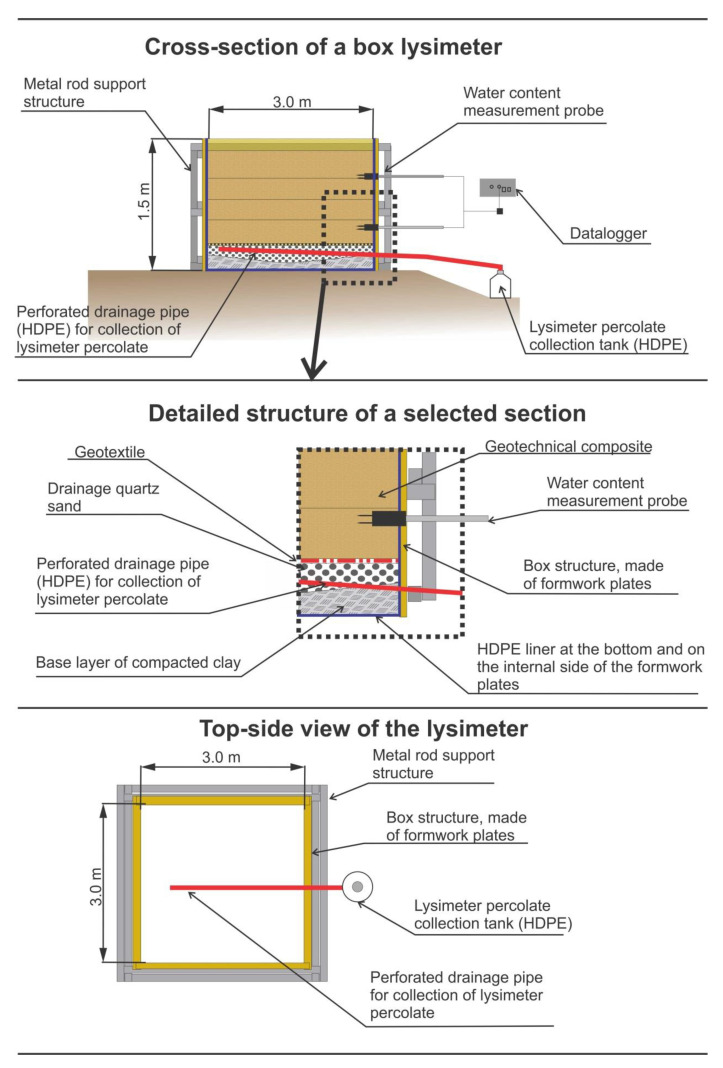
Schematically presented structure of individual lysimeter.

Six box-shaped lysimeters were constructed in the field. The lysimeters were positioned at the facilities of Termit in Moravce, central Slovenia (GK coordinates Y: 482,072.37 X: 110,160.84). A schematic of the structure of an individual lysimeter is shown in Figure 2, while the construction process is presented in Figure S2. Composites with the composition in Table 1 were prepared in the field, with the utilization of an industrial-scale waste-processing technology and construction procedures. The stages of the preparation procedure are presented in Figure S3. Each composite was installed in parallel in two lysimeters in different ways. In the first case it was gradually backfilled into the box structure of the lysimeter and each embankment was levelled with the bucket of an excavator. Thickness of obtained uncompacted layers was around 20 cm, while in the second case, the composite was installed in layers, which were compacted with a vibrating plate. After compaction, each compacted layer was around 10-cm-thick. Each lysimeter was covered with a 15-cm-thick layer of quartz aggregate 4/16, placed on top to prevent surface runoff and evaporation of the precipitation [25].

Uncompacted composites represent an installation that is extremely non-compliant with construction requirements and daily practice. The characteristics of such composites are reflected in a low mechanical stability, a high porosity, and a low leaching resistance. Therefore, a greater infiltration of rainwater and, consequently, more leaching was expected. The installation of the geotechnical composites using compaction was the correct procedure in the earth structures.

The degree of compaction was determined using a nuclear density probe (Troxler 3400, Research Triangle Park, NC). For composites 1 and 2, this parameter was lower than for the laboratory samples, which had at least 98% of the reference dry density. It was not possible to achieve such a high degree of compaction in the lysimeters because only light compaction equipment could be used, due to the limited amount of access.

The dimensions, masses, degree of compaction and permeability of uncompacted and compacted composites installed in the lysimeters are presented in Table 2.

A weather station with a rain gauge, an air-temperature and a humidity probe were installed next to the lysimeters to gather the meteorological data needed to assess the water balance in the lysimeters. Additionally, two sensors for measurements of water content and temperature inside the composites were horizontally installed in each lysimeter, gathering the data every hour.

Percolated water from each lysimeter was collected in a plastic tank of 1000 litres, from which the percolates were taken twice per month, on average, depending on the amount and frequency of precipitation events. The entire volume of collected percolates was measured and poured out from the collector tank at each sampling interval. The data provided in the Supplementary Materials show the amount of precipitation and the volume of percolated water collected between samplings (Table S3a–c), while the fluctuations of the temperature and the moisture content in the composites (upper and lower sensors) are presented in Figure S4a–c. The data points in Figure S4a–c are presented as the average values, which were calculated from the data collected by the temperature and humidity probes between two sampling intervals. As expected, changes in temperature and precipitation reflected seasonal variations. Composites 1 and 3 had better water-retention capacities than Composite 2, in which lower average moisture contents were determined due to the more rapid percolation of water. There were no significant trends in moisture content for the uncompacted and compacted composite 2; the uncompacted composite 1 had a higher moisture content, indicating a higher retention capacity, in comparison to its compacted form; while for composite 3 the situation was the opposite (Figure S4a–c).

### 2.3. Analytical Methods

#### 2.3.1. Determination of Total Element Concentrations of Potentially Toxic Elements in Raw Materials and Composites

To determine the total element concentrations in the raw materials and the laboratory samples of the composites, 0.25 g of lyophilised sample was subjected to microwave-assisted digestion (MARS 6 Microwave System, NC, USA), using a mixture of nitric, hydrochloric and hydrofluoric acids [27], and concentrations of potentially toxic elements (PTEs): cadmium (Cd), lead (Pb), copper (Cu), zinc (Zn), chromium (Cr), nickel (Ni), arsenic (As), selenium (Se), antimony (Sb), molybdenum (Mo), mercury (Hg) and barium (Ba) were determined by means of inductively coupled plasma mass spectrometry (ICP-MS, 7700x, Agilent Technologies, Tokyo, Japan).

#### 2.3.2. Determination of the Mineralogical Composition of the Raw Materials and Composites

The mineralogical compositions of the raw materials and the laboratory samples of the composites were determined by X-ray powder diffraction (XRD), using a PAN analytical diffractometer, with Cu–Kα irradiation (λ = 1.54056 Å), at 45 kV and a current of 40 mA, over the 2θ angular range from 5° to 70°, using a step size of 0.01° and a measuring time per step of 100 s. The patterns were analysed with PAN analytical XʼPert High Score Plus software.

#### 2.3.3. Determination of the Chemical Composition of the Aqueous Leachates and Percolated Water from the Lysimeters

Samples of aqueous leachates from the laboratory tests were filtered through 0.45-µm membrane filters and the total concentrations of PTEs were determined by ICP-MS, according to the procedure EN ISO 17294-2:2016 [28] under the optimal measurement conditions listed in Table S4. The concentrations of chloride (Cl^−^), fluoride (F^−^) and sulphate (SO_4_^2−^), were determined by spectrophotometry (HACH DR/2010, CO, USA), the Cl^−^ and SO_4_^2−^ according to the procedure ISO 15923-1:2013 [29], while F^−^ was determined with the SPADNS method [30]. The phenolic index was determined according to the ISO 14402:1999(E)-point 4 procedure [31]. In each sample, the pH and conductivity were measured (SevenCompact pH/Cond S213, Mettler-Toledo, Columbus, OH, USA). The accuracies of the analyses are listed in Tables S4–S8.

In the laboratory investigations, leaching of the PHSs, given as the concentration (*c*) of each parameter from the dry mass unit of a certain composite, were calculated by multiplying the measured concentration of each individual parameter in the leachate (*a*) by the volume of the leaching solution (*V*), divided by the dry mass (*m_d_*) of a sample of composite, using Equation (1).

(1) $$c\left(mg/kg\right)=\frac{a\;\left(mg/L\right)\;*\;V\;\left(L\right)}{m_{d}\;\left(kg\right)}\;$$

In the case of lysimeter experiments the samples of percolated water were, upon collection, prepared and analysed according to the same protocol as described for the case of the laboratory leachates. Afterwards, the leaching of the PHSs was first calculated for all the sampling intervals. The measured concentration of each individual parameter in the percolated water (*a_i_*) was multiplied by the volume of water percolated in each interval (*V_i_*) and divided by the dry mass of a composite installed in the lysimeter (*m_dl_*), to calculate the concentrations of each parameter (*c_i_*) in each interval. Then the average concentrations of the parameters for all 17 sampling intervals were calculated. The cumulative mass-release concentrations (*C_i_*) of the selected PHSs were calculated for each of 17 successive sampling intervals (*i*) using Equation (2).

(2) $$C_{i}\;\left(mg/kg\right)=\sum_{i=1}^{17}\left(\left(\frac{a_{1}*V_{1}\;}{m_{dl}}\right)+\ldots +\frac{a_{i}*V_{i}\;}{m_{dl}}\right)$$

The trends for the cumulative mass release of selected PHSs were plotted as a function of the cumulative mass-release concentration against the L/S, which increased with time as the cumulative amount of percolated rainwater was also increasing.

## 3. Results and Discussion

### 3.1. Mineralogical Characterization

The mineralogical compositions of the raw materials are shown in Figure S5. The raw materials are composed of crystalline mineral phases. In PMS, PA, CA and FSL amorphous phases are also present, represented in the XRD spectrum as a hump. The elevated background in the XRD spectrum of PMS reflected the presence of an organic component, in which cellulose (partially in crystalline form) predominated (Figure S5). The amorphous humps in the XRD spectres of PA, CA and FSL were assigned to the inorganic glassy phase.

**Figure 3:**
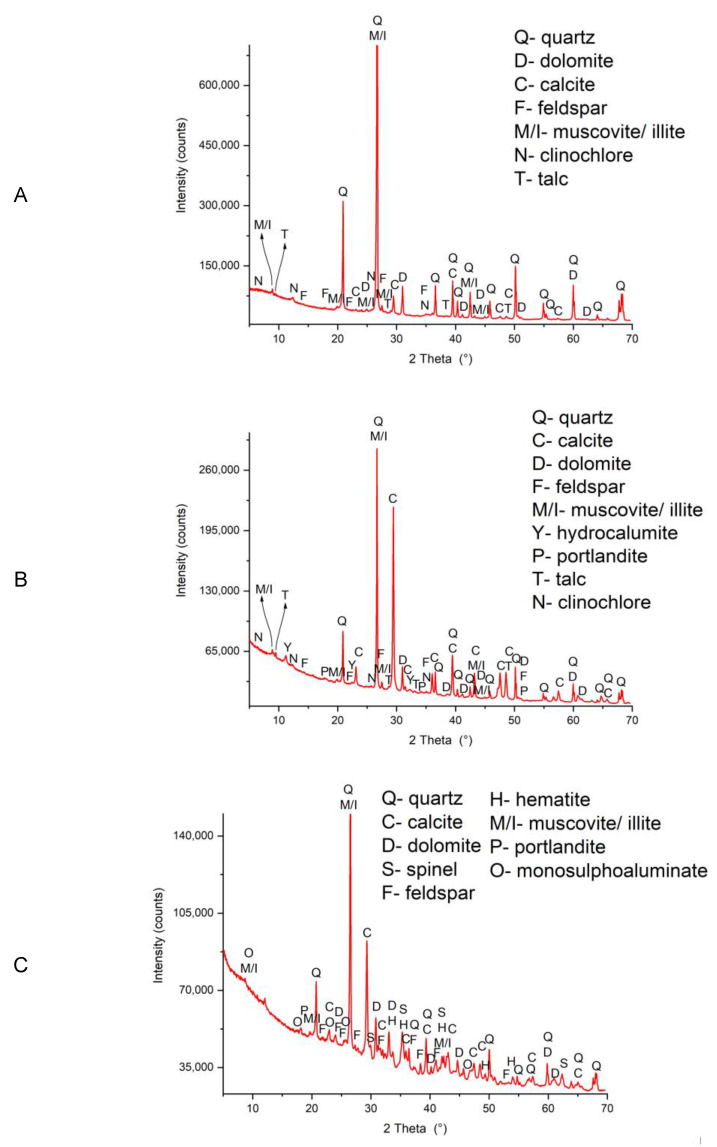
XRD patterns of: (**A**) composite 1; (**B**) composite 2; and (**C**) composite 3.

The mineralogical compositions of the composites are presented in Figure 3.

As is evident from Figure 3, the prevailing mineral phase in composite 1 was quartz, which was also the major crystalline phase of its two constituents: FS and MW (Figure S5). Calcite, kaolinite, talc, and dolomite, which are present as minor phases, originated from the PMS (Figure S5).

In composite 2, quartz and calcite were predominant phases (Figure 3), originating from all three raw materials of the composite (Figure S5). Among the minor phases, dolomite, feldspar, muscovite/illite, talc and clinochlore were introduced into the composite with the addition of DI and MW, while portlandite originated from the PA (Figure S5). The newly formed mineral phase in composite 2 was identified in the XRD spectra as hydrocalumite (Figure 3). It is a result of the pozzolanic and hydration reactions, which are characteristic for PA [9,22].

In composite 3, quartz was the predominating crystalline constituent, which originated from the CA, SWIA and FSL (Figure S5). Other mineral phases were present in a minority, for example, hematite and spinel (from SWIA) (Figure S5). In composite 3, a newly formed mineral phase, i.e., mosoluphoaluminate, was identified, which was the hydration and pozzolanic product from the use of CA (Figure 3).

### 3.2. Total Concentrations of PTEs in the Raw Materials and Composites

The total concentrations of PTEs in the raw materials and composites after microwave-assisted digestion determined by ICP-MS are presented in Tables S9 and S10, respectively.

As evident from Table S9, the total concentrations of PTEs in the raw materials were the highest in bottom ash from the solid-waste incineration and in the foundry sand. The comparison of the total concentrations and the concentrations of these elements in aqueous leachates (Table S11) showed that their mobility was very low, although some values exceeded the national limits for end-of-waste criteria [32].

### 3.3. Environmental Impacts of Geotechnical Composites

To evaluate the environmental acceptability of the geotechnical composites, laboratory and field experiments were performed, as described in the experimental set-up (2.4).

#### 3.3.1. Leaching of Potentially Hazardous Substances from the Raw Materials and Laboratory Samples of Geotechnical Composites

**Figure 4:**
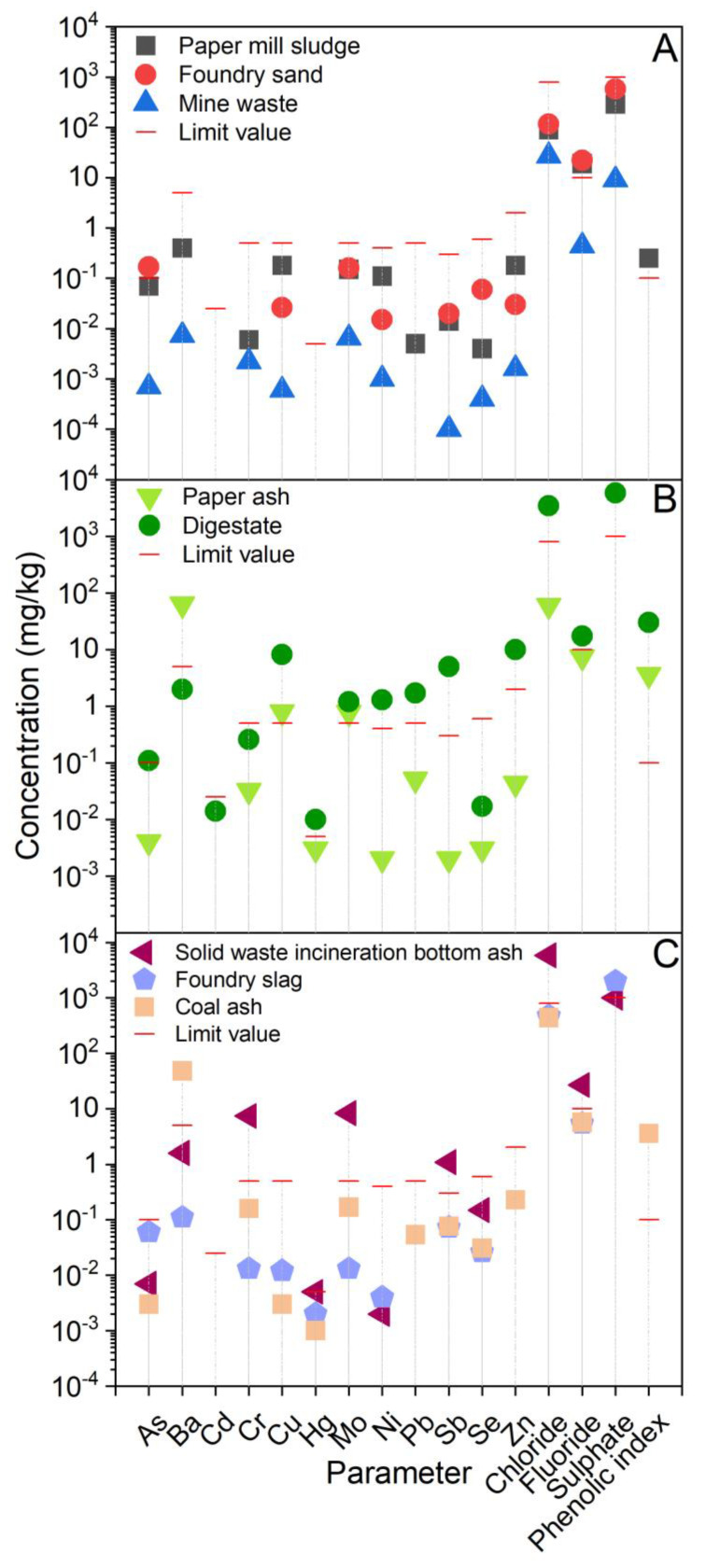
Concentrations of potentially hazardous substances in aqueous leachates from raw materials: (**A**) paper-mill sludge, foundry sand and mine waste; (**B**) paper ash and digestate; and (**C**) slag from waste incineration, foundry slag and coal combustion ash, used for the preparation of geotechnical composites. Limit values (from the Decree on waste; Official Gazette of Republic of Slovenia, 2020) are also given.

**Figure 5:**
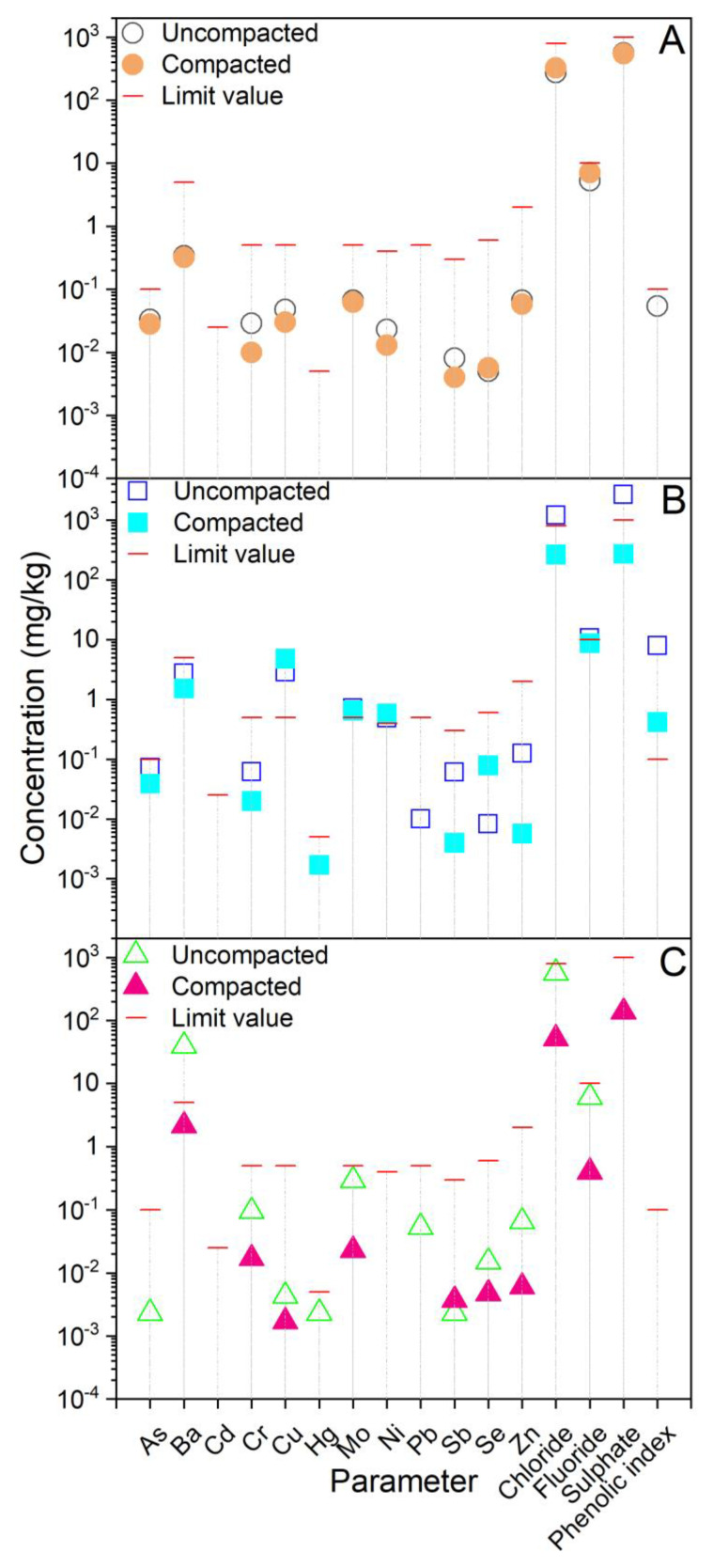
Concentrations of potentially hazardous substances in aqueous leachates from uncompacted and compacted geotechnical composites: (**A**) composite 1; (**B**) composite 2; and (**C**) composite 3. Limit values from the Decree on waste; Official Gazette of Republic of Slovenia, 2020 are also given.

Aqueous leachates from the raw materials and the laboratory samples of composites are presented in Figure 4 and Figure 5, respectively, while the pH values, conductivity as well as the individual concentrations of the elements and anions related to the data from Figure 4 and Figure 5, are provided in Tables S11 and S12.

The data presented in Figure 4 show that in all the raw materials, the limits were exceeded for at least one parameter, when considering the Slovenian end-of-waste criteria [32]. The exception was MW, in the leachates of PMS and FS, where the concentrations of F^−^ were around twice the limit. In addition, As in the leachate of FS was nearly twice the limit. In PA, Ba greatly exceeded the limit (by a factor of 12), while Cu and Mo exceeded this value by 1.5 times. In the leachate from DI, concentrations of most parameters (i.e., As, Cu, Hg, Mo, Ni, Pb, Sb, Zn and all anions) exceeded the limits. Cu and Sb exceeded the limits by a factor of 16, and Zn and Cl^−^ by a factor of 5. In the leachate of SWIA, the concentrations of Cr, Hg, Mo, Sb and all anions exceeded the limits, Cr and Mo by 16 times and Cl^−^ by 7 times. In FSL, only SO_4_^2−^ exceeded the limit, by 2 times, while in CA, the Ba in the leachate exceeded the limit by a factor of 10.

The results of the leaching tests from Composites 1, 2 and 3 are presented in Figure 5, while the support data are summarized in Table S12.

Composite 1 (Figure 5A) fulfilled the environmental acceptability criteria in both the uncompacted and compacted forms. Composite 1 produced a slightly alkaline leachate (pH 8). PMS and MW are considered as immobilization additives due to the constituents having a high sorption capacity (clay minerals) [33]. Despite the presence of phenolic polymer resins in the FS, the values of the phenolic index in the leachate were below the determination limit.

Legislative criteria were also met for composite 3 (Figure 5C) in the compacted form. In composite 3, the CA was used as a pozzolanic and hydraulically active alternative binder. The binding ability of the CA was evident from the formation of a new phase from the group of CAH minerals (i.e., monosulfoaluminate—Ca_4_Al_2_SO_10_ 16H_2_O) (Figure 3), which is also known to be able to incorporate soluble ions of PTEs in its crystalline lattice and chemically immobilize them [34]. Lime and portlandite are the constituents of the CA that produced an alkaline pH of the pore solution in composite 3, which favoured the formation of insoluble or low-soluble metal hydroxides.

In the uncompacted composite 3, only Ba did not meet the legislative criterion. The reason is that in alkaline conditions, the highly soluble Ba hydroxide was formed, and not enough soluble sulphate was available in the pore solutions to lower its solubility through the formation of the low-solubility Ba-sulphate [35]. On the other hand, in the compacted composite the Ba ions were chemically immobilized because of the effective diffusion of the ions to the surface adsorption sites of the clay minerals from MW. Compacted composite 3 had a low water permeability, also enabling the effective physical immobilization of Ba [9,22].

In composite 2 (Figure 5B) the environmental limits were exceeded for parameters Cu, Mo, and Ni, as well as for the phenolic index in both the uncompacted and compacted forms, and additionally for SO_4_^2−^, Cl^−^ and F^−^ in the uncompacted form. High values of the phenolic index indicated the presence of polyphenolic compounds, which were evolving either from the humic and fulvic substances, or due to their formation during the microbiological decay of the plastic waste in the mechanical-biological treatment, in which the DI was generated [36]. In this composite, PA had the role of an alternative binder and the main immobilization additive because of its pozzolanic and hydraulic activity [37]. By mixing humid DI with dry PA, instant hydration of the CaO (lime) present in the PA occurred, resulting in the formation of portlandite (Ca(OH)_2_) and a pore solution saturated with Ca^2+^ and OH^−^ (Figure 3). High pH values of the pore solutions in the composite had perturbing effects on the microbial community [38,39]. In this way, pathogenic organisms potentially present in the DI after the microbiological treatment of this waste were destroyed (see pH and electrical conductivity in leachate from composite 2, Table S12). The binding characteristics of the PA were reflected in the formation of the hydration product, i.e., calcium aluminate hydrate (CAH), more specifically hydrocalumite (Ca_4_Al_2_(OH)_12_(Cl,CO_3_,OH)_2_ 4H_2_O). The CAH present in composite 2 chemically immobilized the PTEs by incorporating them into its stable crystalline lattice [39]. However, Cu and Ni were already strongly complexed with soluble organic matter (e.g., humic and fulvic acids, and low-molecular-mass organic acids) [40,41], and therefore remained mobile. The leaching of Cu was much higher than that of Ni, because Cu formed stronger complexes with the organic matter and its total content in the composite was 10 times higher [40,42]. In the uncompacted composite, the conditions were aerobic, which resulted in a lower pH (due to carbonation reactions) and therefore the mobility of the dissolved organic matter was lower than for the anaerobic conditions in the compacted composite [43]. Accordingly, the pH of the leachate from the uncompacted composite 2 was lower (pH 10.8) than in the compacted sample (pH 11.6) (Table S12). Due to the smaller amount of dissolved organic matter, the extent of the Cu and Ni complexation and thus the Cu and Ni release from the uncompacted composite 2 was smaller than from the compacted sample (Table S12) [44]. The release of Mo from composite 2 was related to the negative charge of the molybdate oxyanion (MoO_4_^2−^), which was not effectively immobilised by the constituents of composite 2 at the highly alkaline pH. The slightly lower leaching of Mo from the compacted composite was due to physical immobilization. Similarly, negatively charged SO_4_^2−^, Cl^−^ and to a lesser extent F^−^, were not effectively immobilized under the highly alkaline conditions in the uncompacted composite 2. However, in the compacted form, the leaching of these anions was significantly reduced below the legislative limits due to physical immobilization.

#### 3.3.2. Results from the Field Lysimetric Experiments

The mobility of PHSs from the geotechnical composites installed in the lysimeters was followed from 10th June 2020 to 3rd June 2021.

The difference in the results between the laboratory leaching tests and the results from the lysimeter tests was expected, and they were in line with the literature reports [12,13]. In laboratory, under such water-saturated conditions, the chemical equilibria were achieved during leaching due to a surface dissolution and diffusion of the PHSs [44]. In the lysimetric tests, rainwater was the leaching solution, which was infiltrated and gradually percolated through the geotechnical composites under sub-saturated conditions [13,44]. The cumulative L/S ratio in the lysimeters was more than 10-times lower than for the laboratory test, only from 0.6 to 1.1 for the uncompacted composites, and 0.4 to 0.9 for the compacted composites. The lower L/S ratio for the compacted composites was due to the lesser infiltration of rainwater due to their lower porosity.

The pH and the conductivity of the laboratory leachates from composites 1 and 2 were higher than the average values determined in the percolates. Lower pH values in the percolates were due to the more intensive carbonation but can also be attributed to the hydrodynamics of the rainwater infiltration, which can cause the localized wash-out of alkalis, among other ions, when probably preferential leaching routes were formed. Slightly lower pH values and the higher conductivity of the laboratory leachates in comparison to lysimeter percolates were determined for composite 3. This can be attributed to the slower carbonation and the diffusion of alkalis from this composite at lower L/S ratios in comparison to the laboratory tests [12].

Time-dependent changes in the conductivity and the pH values of the percolates are presented in Figures S6 and S7. The pH values of the percolates from composite 1 remained relatively constant during the field experiment, while a slight decrease was observed in composites 2 and 3, which is related to the carbonization. Due to a more intensive wash-out in composite 3 the pH values were higher in the uncompacted composite. This is also indicative of the high concentration of alkalis in the CA. The conductivity of the percolates gradually decreased with time due to the continuous leaching and the wash-out of ions, and due to the gradual time-dependent increase in their immobilization and the formation of new mineral phases, especially in composites 2 and 3, in which pozzolanic and hydraulically active ashes were used [22].

The results for the different parameters, given as an average value for all the concentrations determined in the percolates from all the sampling intervals, are presented in Figure S8, while the support data are given in Table S3a–c.

The lowest average concentrations of PHSs for all the sampling intervals were determined in percolates from the compacted composites 1 and 3. In composite 2, very low average concentrations of PHS were also determined in the percolates, with the exception of Cu, where the concentration was about 4500 times higher than in Composites 1 and 3, similar to the laboratory experiments.

The average leaching of PHSs from the geotechnical composites in the field was significantly lower (generally 25 to 150 times), compared to the leaching from the composites in the laboratory tests. This difference cannot be solely attributed to the difference in L/S, but also indicates that the same immobilization mechanisms of PHSs, as discussed in Section 3.3.1, took place in composites in the lysimeters, but were more effective under natural conditions and over a longer time period, in the case of the field experiment, in comparison to the short-term laboratory experiments [45].

**Figure 6:**
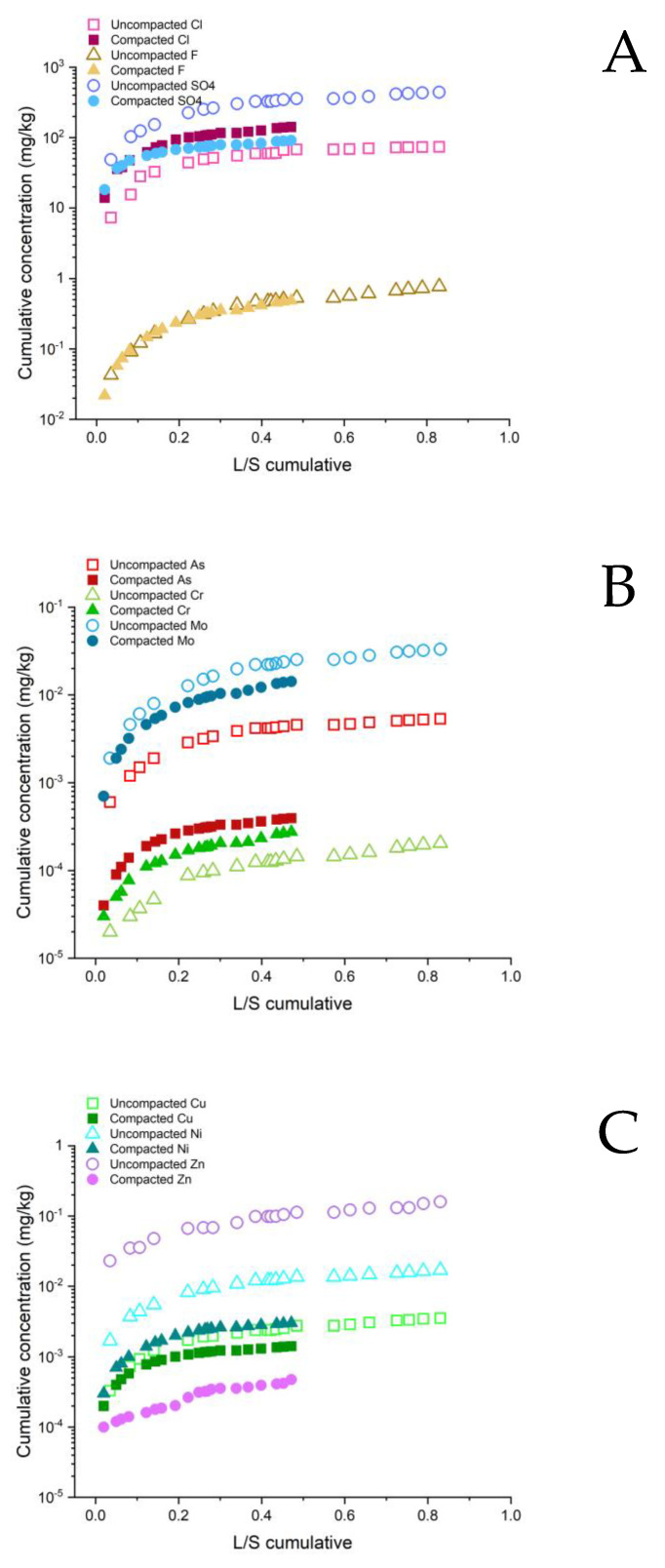
Cumulative concentrations of: (**A**) Cl^−^, F^−^, SO_4_^2−^ (mg/kg); (**B**) As, Cr, Mo (mg/kg); and (**C**) Cu, Ni, Zn (mg/kg) as a function of cumulative liquid to solid ratio (L/kg) in field lysimetric experiments in uncompacted and compacted composite 1.

**Figure 7:**
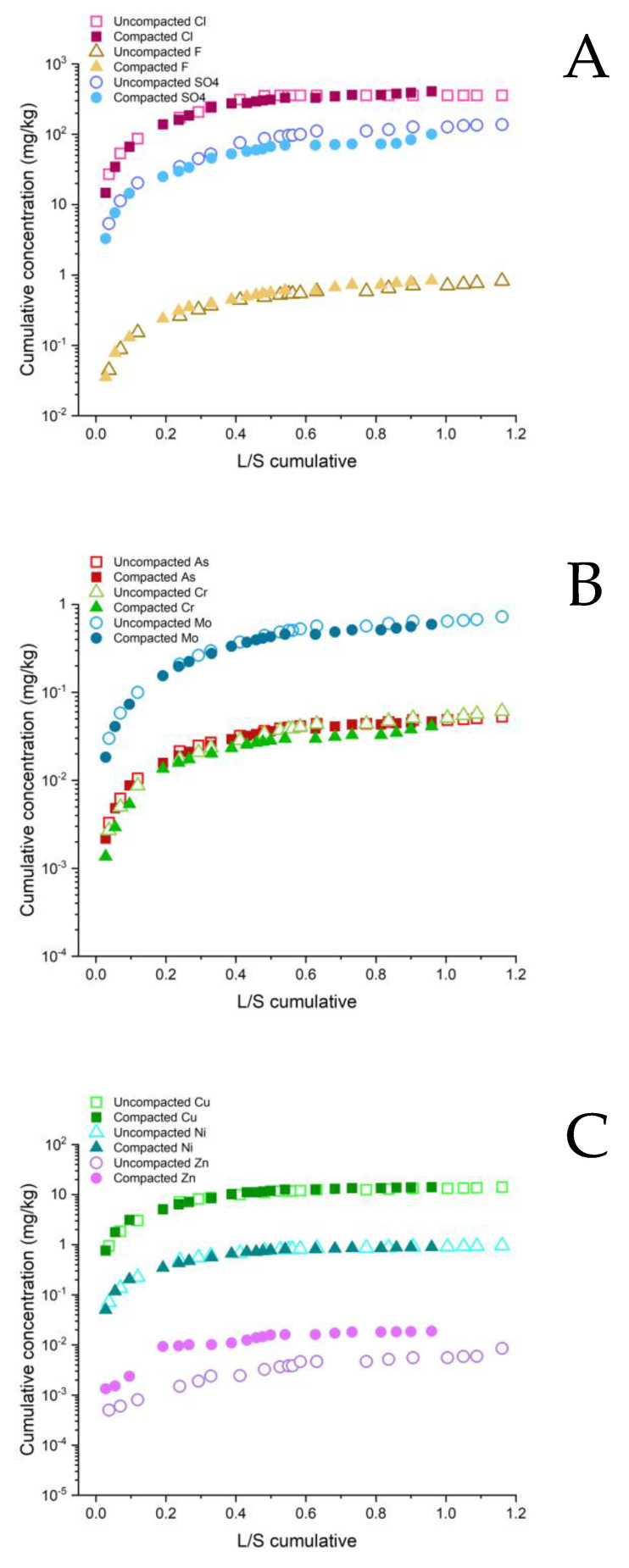
Cumulative concentrations of: **(A)** Cl^−^, F^−^, SO_4_^2−^ (mg/kg); (**B**) As, Cr, Mo (mg/kg); and (**C**) Cu, Ni, Zn (mg/kg) as a function of cumulative liquid to solid ratio (L/kg) in field lysimetric experiments in uncompacted and compacted composite 2.

**Figure 8:**
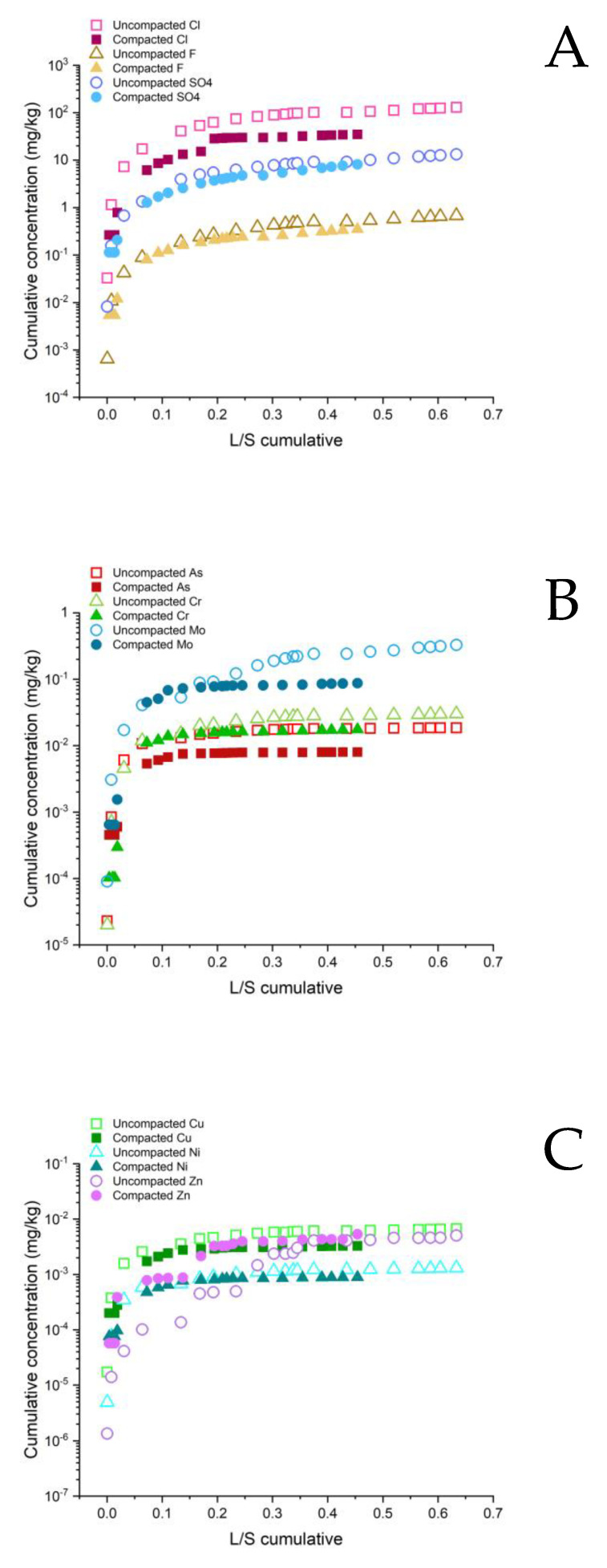
Cumulative concentrations of: (**A**) Cl^−^, F^−^, SO_4_^2−^ (mg/kg); (**B**) As, Cr, Mo (mg/kg); and (**C**) Cu, Ni, Zn (mg/kg) as a function of cumulative liquid to solid ratio (L/kg) in field lysimetric experiments in uncompacted and compacted Composite 3.

Figure 6, Figure 7 and Figure 8 show a comparison between the trends in cumulative time-dependent mass releases of selected PHSs in relation to the L/S value. A larger amount of rainwater percolated through the uncompacted composites 1 and 3 compared to their compacted forms. Consequently, a higher cumulative mass release of PHS came from the uncompacted composites. This proved that when the composites were compacted up to the maximum dry density the effective physical immobilization of the PHSs can be achieved [14]. The only exceptions, with a slightly higher concentration in the compacted form, were Cl^−^ and Cr in composite 1. The infiltration and percolation of the water in composite 2 were very similar for the compacted and uncompacted forms. This was also reflected in the mass release of the PHSs, which was almost the same in both cases. The exception was Zn, which showed more intensive leaching from the compacted composite. This can be attributed to the fact that higher dissociation and crushing of the grains occurred during the installation of the composite by compaction, which led to the release of Zn [46]. The results from the lysimeter were not fully comparable with the observations from the laboratory experiments, in which more intensive leachings of the elements (Mo, Cu, and Ni) were determined in the compacted composite, which was probably due to the heterogenetic composition of DI or due to the more intensive crushing of contaminated grains during the compaction in the laboratory [46].

Higher values of the cumulative mass releases of As, Cr, and Mo were determined for composites 2 and 3, even though the total content of these elements was much lower in comparison to composite 1. This might be related to the lower immobilization of these oxyanion metals due to alkaline pore solutions in both composites or due to complexation with soluble organic matter in composite 2 [44,47]. The immobilization of Cu, Ni, and Zn appeared to be the highest in composite 3 due to the formation of low-solubility hydroxides and incorporation in the newly formed mineral phases [48], followed by composite 1, while the least effective was in composite 2, in which the leaching of these metals was increased due to the complexation with dissolved organic matter that has increased mobility under alkaline conditions in this composite [42,48,49].

The trends in the cumulative mass release of the PHSs, as seen in the logarithmic scale graphs, indicated a sharp increase in the initial time intervals after the installation, followed by a steady decrease in the intensity of the leaching in the later time intervals. Cumulative values for composite 3 appeared to stabilize due to effective the immobilization. The intensity of the long-term release of the PHSs is relatively low and stable. Exceptions are SO_4_^2−^, F^−^ (uncompacted and compacted composite 3) and Mo (uncompacted composite 3), in which the intensity of long-term release is relatively higher compared to other PHSs. In contrast, for composite 1, the slightly increasing trends in cumulative mass release can be observed for all the parameters in Figure 6, indicating that a certain mass flow of PHSs can be expected in the future, but it appears that the immobilization is effective. Similar trends were observed for composite 2 (Figure 7); however, the immobilization in this composite was far less effective, and therefore the cumulative mass release of PHSs was more intensive in comparison to composites 1 and 3.

## 4. Conclusions

The environmental properties of three geotechnical composites, made of various voluminous waste materials, were studied on the basis of laboratory and field investigations. The wastes used had concentrations of PHS in aqueous leachates that exceeded the threshold values set by the national legislation.

After being processed into composites, immobilization of the PHSs occurred. The results obtained in the standardised laboratory leaching tests showed that composite 1 and composite 3 complied with the Slovenian limits for end-of-waste status. Meanwhile, in the leachate from composite 2 the parameters of Cu, Mo and Ni exceeded the limits.

The composites were installed in lysimeters in uncompacted and compacted forms to investigate the influence of the installation process on the environmental properties. The leaching of the PHSs was studied. More effective immobilization of the PHSs was observed in the field lysimeters than in the laboratory experiments. This was attributed to the longer period of exposure to natural conditions. The average concentrations of PHSs in the percolated water from compacted composites were lower in comparison to the uncompacted ones. After one year of monitoring, the trends in the cumulative mass release of the PHSs decreased in the intensity of leaching observed for composite 3, which was considered to have achieved an effective long-term immobilization. For composite 1 and composite 2, increasing trends of the cumulative mass release of the substances persisted, but decreased with time; the composites were considered to have achieved both a progressive immobilization and the wash-out of the PHSs. After a longer time, the PHSs also slowly diffused through the composites and bonded to the surface adsorption sites on the clay minerals from the MW. Pozzolanic and hydration reactions that took place in composite 2 and composite 3, due to the addition of ashes, resulted in higher immobilization efficiency due to the formation of larger quantities of newly formed mineral phases that incorporated the PHS into their stable crystalline structure. The lowering trend of the pH values of the percolate from uncompacted composite 2 indicated that a process of carbonation took place in the composites in which the ashes were used. The drop in the pH values that was observed for composite 3 was only minor, due to a high buffer capacity.

Based on the results of this study it can be concluded that composite 1 and composite 3 do not represent an environmental hazard, over the long term and under natural conditions. Therefore, they can be used for the rehabilitation of abandoned open-pit mines, while the use of composite 2 is limited, for example, to closure operations at a landfill for non-hazardous wastes, where potential leaching of the PHSs into the environment can be prevented.

This study confirmed the need for a holistic approach to studying the environmental properties of recycled materials from construction products. Such an approach can be a basis for the successful transfer of recycling solutions that are developed in the laboratory to the field.

## Figures and Tables

**Table 1:** Composition of the geotechnical composites.

Raw Material	Composite 1 (wt.%)	Composite 2 (wt.%)	Composite 3 (wt.%)
Paper-mill sludge (PMS)	20	/	/
Foundry sand (FS)	30	/	/
Mine waste (MW)	50	20	50
Paper ash (PA)	/	40	/
Digestate (DI)	/	40	/
Solid-waste incineration bottom ash (SWIA)	/	/	5
Foundry slag (FSL)	/	/	5
Coal ash (CA)	/	/	40

**Table 2:** Selected geometrical and technical parameters geotechnical composites installed in the lysimeters and their permeability expressed using the infiltration coefficient.

Parameter	Composite 1		Composite 2		Composite 3	
	Uncompacted	Compacted	Uncompacted	Compacted	Uncompacted	Compacted
Surface area exposed to precipitation (m^2^)	9	9	9	9	9	9
Height of a geotechnical fill in lysimeter (m)	1.15	1.15	1.35	1.20	1.20	1.25
Degree of compaction (%) *	n.a.	94	n.a.	92	n.a.	99
Dry mass of installed composite (Mg)	13.45	18.32	9.16	11.52	13.41	15.85
Infiltration coefficient (m/s) **	7.5 × 10^−4^	2.1 × 10^−7^	1.7 × 10^−4^	5.6 × 10^−5^	6.3 × 10^−6^	6.3 × 10^−7^

* Ratio between maximum reference dry density and dry density of installed composite. ** Measured according to the ISO/DIS 22282-5:2007 [26].

## Data Availability

All the data related to the paper are provided in the main manuscript and the Supplementary Materials. Additional data related to this paper may be available from the corresponding author on request.

## Supplementary Materials

The following supporting information can be downloaded at: https://www.mdpi.com/article/10.3390/ijerph20032014/s1, Figure S1: A flow chart of the experimental set-up; Figure S2: Different stages of a field construction of lysimeters: Metal rod support structure (A) and the same structure placed at the final location of lysimeters (B), installation of an internal box structure, made from water resistant formwork plates (C), and the bottom-up view on the finalized structure of the box lysimeters, with a lysimeter percolate collection tank placed in front (D); Figure S3: Different stages of a field preparation of geotechnical composites for the lysimeter experiments: Preparation of a composite, according to predetermined dry mass ratios and with addition of optimal amount of water, by the use of excavator mixing bucket (A), filling of the lysimeter with a layer of a composite (B), spreading of a composite in a layer of uniform thickness of 30 cm (C), and the final installation of the layer of a composite by a vibration compactor in box lysimeters (D); Figure S4a: Temperature (A) and moisture (B) of uncompacted and compacted Composite 1 (measurements from upper and lower sensor); Figure S4b: Temperature (A) and moisture (B) of un-compacted and compacted Composite 2 (measurements from upper and lower sensor); Figure S4c: Temperature (A) and moisture (B) of un-compacted and compacted Composite 3 (measurements from upper and lower sensor); Figure S5: XRD patterns of raw materials (A) Paper mill sludge, (B) Foundry sand, (C) Mine waste, (D) Paper ash, (E) Digestate, (F) Bottom ash from incineration of waste, (G) Foundry slag and (H) Coal ash; Figure S6: pH values of percolates from (A) Composite 1, (B) Composite 2 and (C) Composite 3 as function of different sampling period. Results represent values obtained from samplings in a period from June 2020 to June 2021; Figure S7: Conductivity values (mS/m) of percolates from (A) Composite 1, (B) Composite 2 and (C) Composite 3 as function of different sampling period. Results represent values obtained from samplings in a period from June 2020 to June 2021; Figure S8: Concentrations of potentially hazardous substances in percolated water from lysimeters installed below uncompacted and compacted geotechnical composites (A) Composite 1, (B) Composite 2 and (C) Composite 3. Results represent average concentrations of elements and anions obtained from 18 (Composites 1 and 3) or 17 (Composite 2) samplings in a period from June 2020 to June 2021; Table S1: Basic information about the input raw materials used in the experiments; Table S2: Maximum reference dry density and optimal moisture content of geotechnical composites; Table S3a: Precipitation, volume of collected lysimetric water of PTEs and anions (dry mass basis), pH and conductivity in lysimetric water from uncompacted Composite 1; Table S3b: Precipitation, volume of collected lysimetric water of PTEs and anions (dry mass basis), pH and conductivity in lysimetric water from uncompacted Composite 2; Table S3c: Precipitation, volume of collected lysimetric water of PTEs and anions (dry mass basis), pH and conductivity in lysimetric water from uncompacted Composite 3; Table S4: ICP-MS (Agilent 7900) operating parameters for the determination of element concentrations in aqueous leachates; Table S5: Certified and determined concentrations of PTEs in the standard reference material SPS-SW1 (Reference material for measurements of elements in surface waters) determined by ICP-MS. The results represent the mean concentration obtained from four parallel samples; Table S6: Concentrations of chlorides, fluorides and sulphates in the standard reference material Anions—While Volume obtained from Merck KGaA (Darmstadt, Germany) determined by spectrophotometry. The results represent the mean concentration obtained from four parallel samples; Table S7: Concentrations of elements in certified reference material CRM 320R (Trace Elements in River Sediment) determined by ICP-MS after microwave-assisted digestion. The results represent the mean concentration from three parallel samples; Table S8: LODs for the determination of element concentrations aqueous leachates and lysimetric waters by ICP-MS and anions by spectrophotometry, and determination of the total element concentrations in composite samples by ICP-MS; Table S9: Total concentrations of PTEs in raw materials after microwave-assisted digestion determined by ICP-MS. The expanded uncertainty of the analytical procedure was better than ± 3% (k = 2); Table S10: Total concentrations of PTEs in composites after microwave-assisted digestion determined by ICP-MS. The results represent the mean concentration obtained from three parallel samples; Table S11: Concentrations of PTEs and anions in aqueous leachates from raw materials applying the SIST EN 1744-3:2002 leaching test. Limits for inertness, set by the current Slovenian legislation, are also given. Values highlighted with red exceed the threshold values for inertness. pH and electrical conductivity of aqueous leachates are also given. The expanded uncertainty of analytical procedure was better than ± 3% (k = 2); Table S12: Concentrations of PTEs and anions in aqueous leachates from three uncompacted and compacted Composites 1, 2 and 3, applying SIST EN 1744-3:2002 leaching test. Results represent average concentrations of elements and anions obtained from three analysed samples for each composite. Limits for inertness, set by the current Slovenian legislation, are also provided. Values highlighted with red exceed the threshold values for inertness. pH and electrical conductivity of aqueous leachates are also given. The expanded uncertainty of analytical procedure was better than ± 3% (k = 2).
