# Supplementary material for: Environmental Acceptability of Geotechnical Composites from Recycled Materials: Comparative Study of Laboratory and Field Investigations
Source: Int J Environ Res Public Health. 2023 Jan 21;20(3):2014. doi: 10.3390/ijerph20032014 (PMC9915455; doi:10.3390/ijerph20032014)
Supplement: Supplementary file 1 [file ijerph-20-02014-s001.zip › Figure s2.pdf]

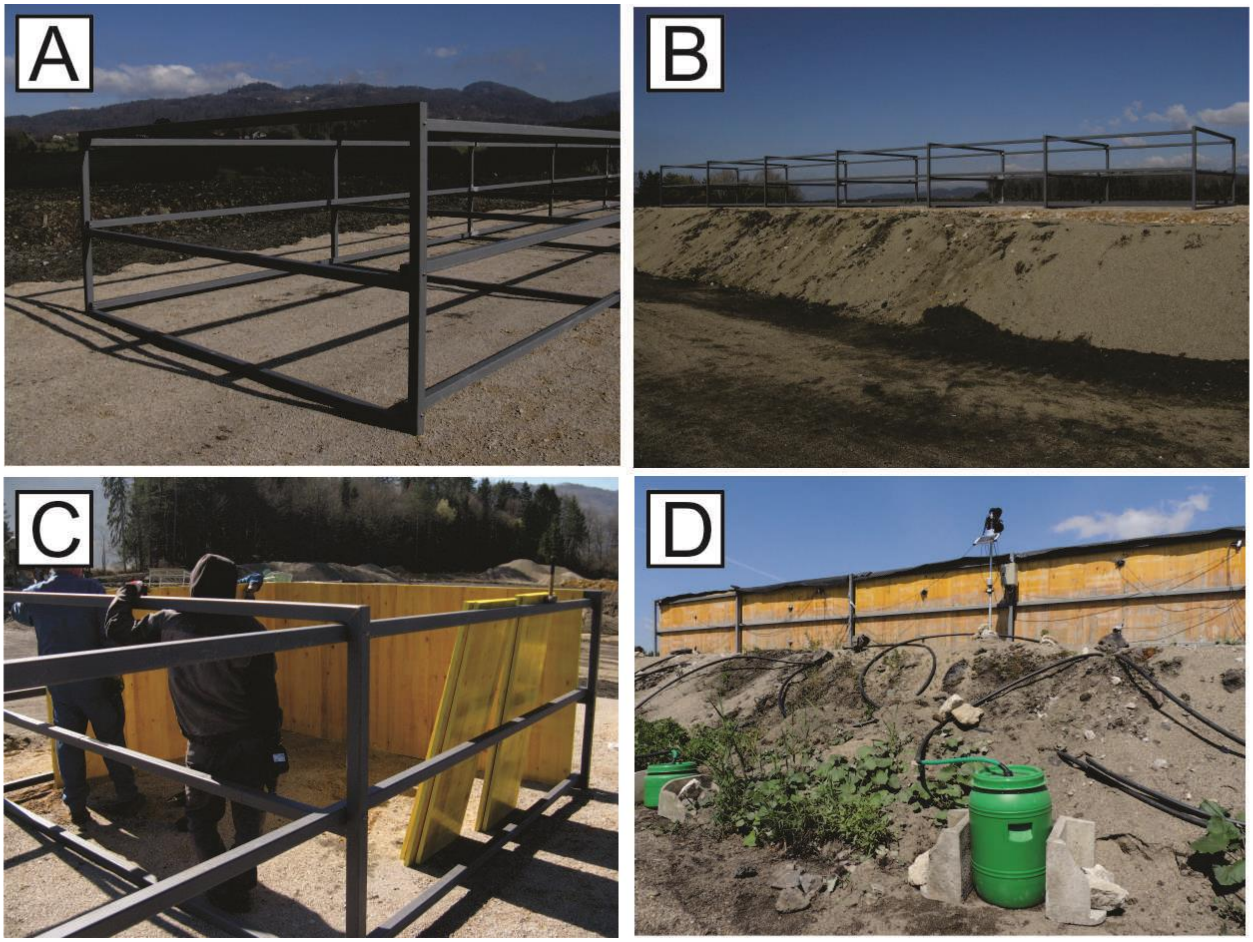

Figure S2 Different stages of a field construction of lysimeters: Metal rod support structure (A) and the same structure placed at the final location of lysimeters (B), installation of an internal box structure, made from water resistant formwork plates (C), and the bottom-up view on the finalized structure of the box lysimeters, with a lysimeter percolate collection tank placed in front (D).
