# Supplementary material for: Environmental Acceptability of Geotechnical Composites from Recycled Materials: Comparative Study of Laboratory and Field Investigations
Source: Int J Environ Res Public Health. 2023 Jan 21;20(3):2014. doi: 10.3390/ijerph20032014 (PMC9915455; doi:10.3390/ijerph20032014)
Supplement: Supplementary file 1 [file ijerph-20-02014-s001.zip › Figure s3.pdf]

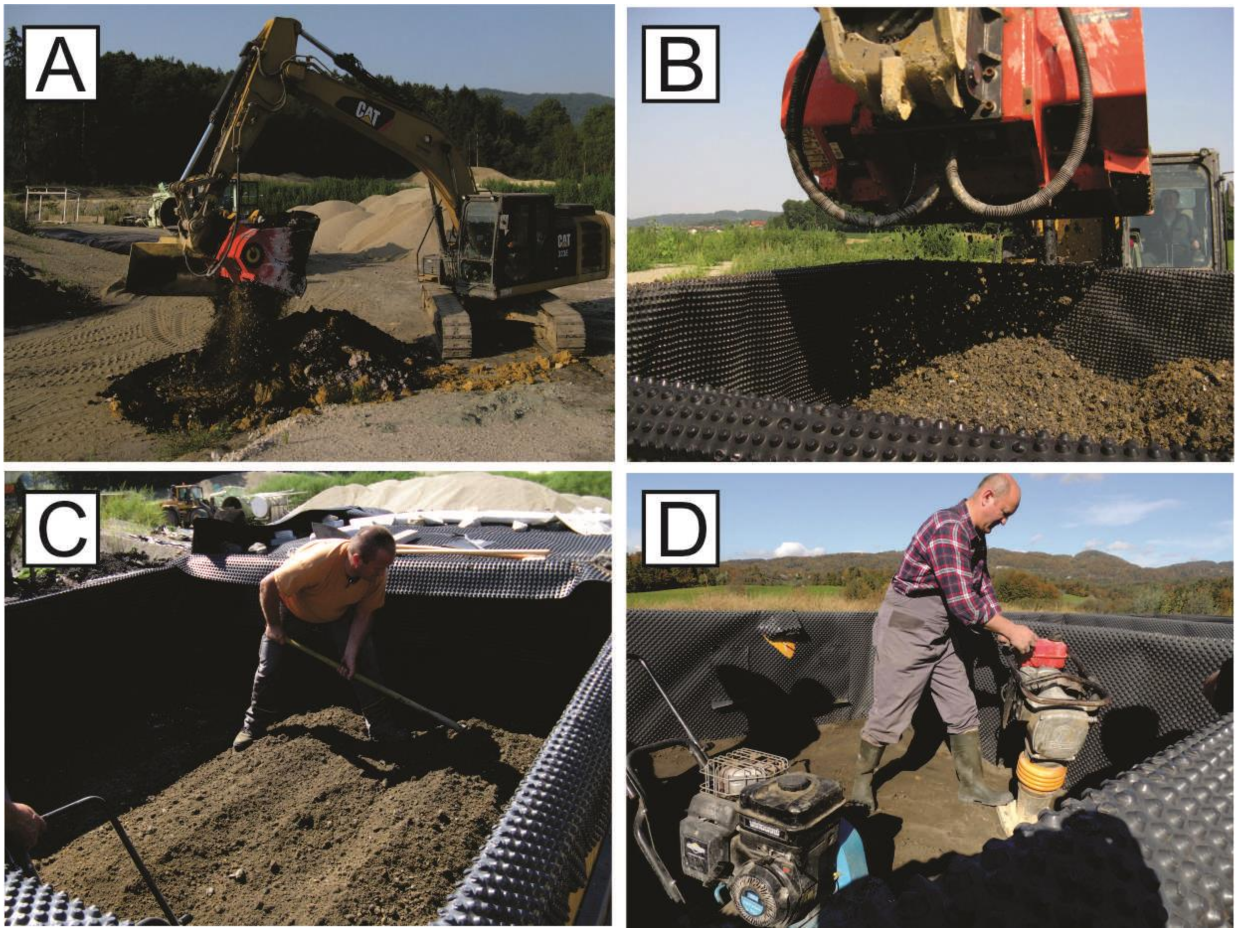

Figure s3 Different stages of a field preparation of geotechnical composites for the lysimeter experiments: Preparation of a composite, according to predetermined dry mass ratios and with addition of optimal amount of water, by the use of excavator mixing bucket (A), filling of the lysimeter with a layer of a composite (B), spreading of a composite in a layer of uniform thickness of 30 cm (C), and the final installation of the layer of a composite by a vibration compactor in box lysimeters (D).
