# Supplementary material for: Environmental Acceptability of Geotechnical Composites from Recycled Materials: Comparative Study of Laboratory and Field Investigations
Source: Int J Environ Res Public Health. 2023 Jan 21;20(3):2014. doi: 10.3390/ijerph20032014 (PMC9915455; doi:10.3390/ijerph20032014)
Supplement: Supplementary file 1 [file ijerph-20-02014-s001.zip › Figure s4C.pdf]

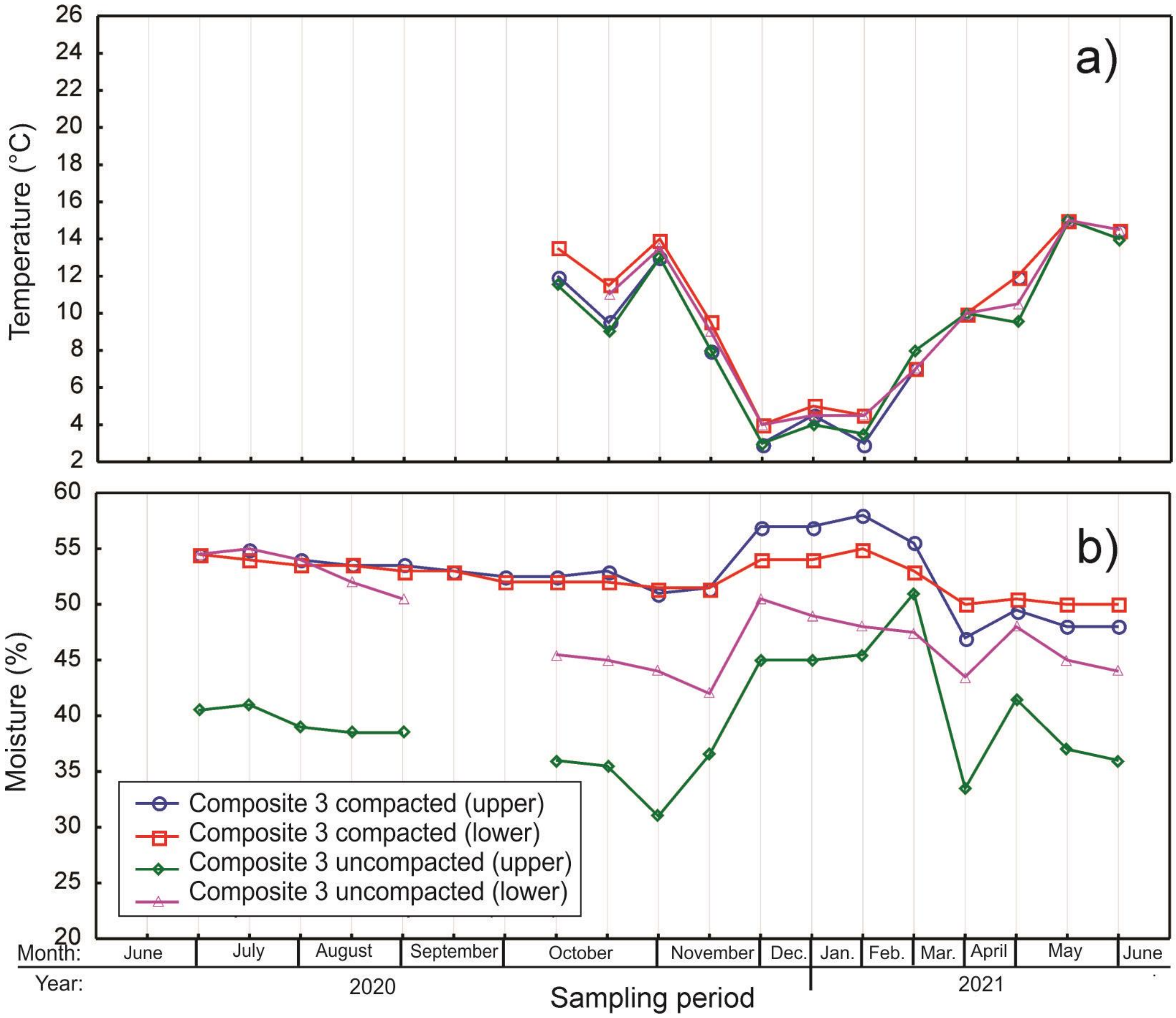

Figure S4c Temperature (A) and moisture (B) of un-compacted and compacted Composite 3 (measurements from upper and lower sensor).
