# Supplementary material for: Environmental Acceptability of Geotechnical Composites from Recycled Materials: Comparative Study of Laboratory and Field Investigations
Source: Int J Environ Res Public Health. 2023 Jan 21;20(3):2014. doi: 10.3390/ijerph20032014 (PMC9915455; doi:10.3390/ijerph20032014)
Supplement: Supplementary file 1 [file ijerph-20-02014-s001.zip › Figure s5.pdf]

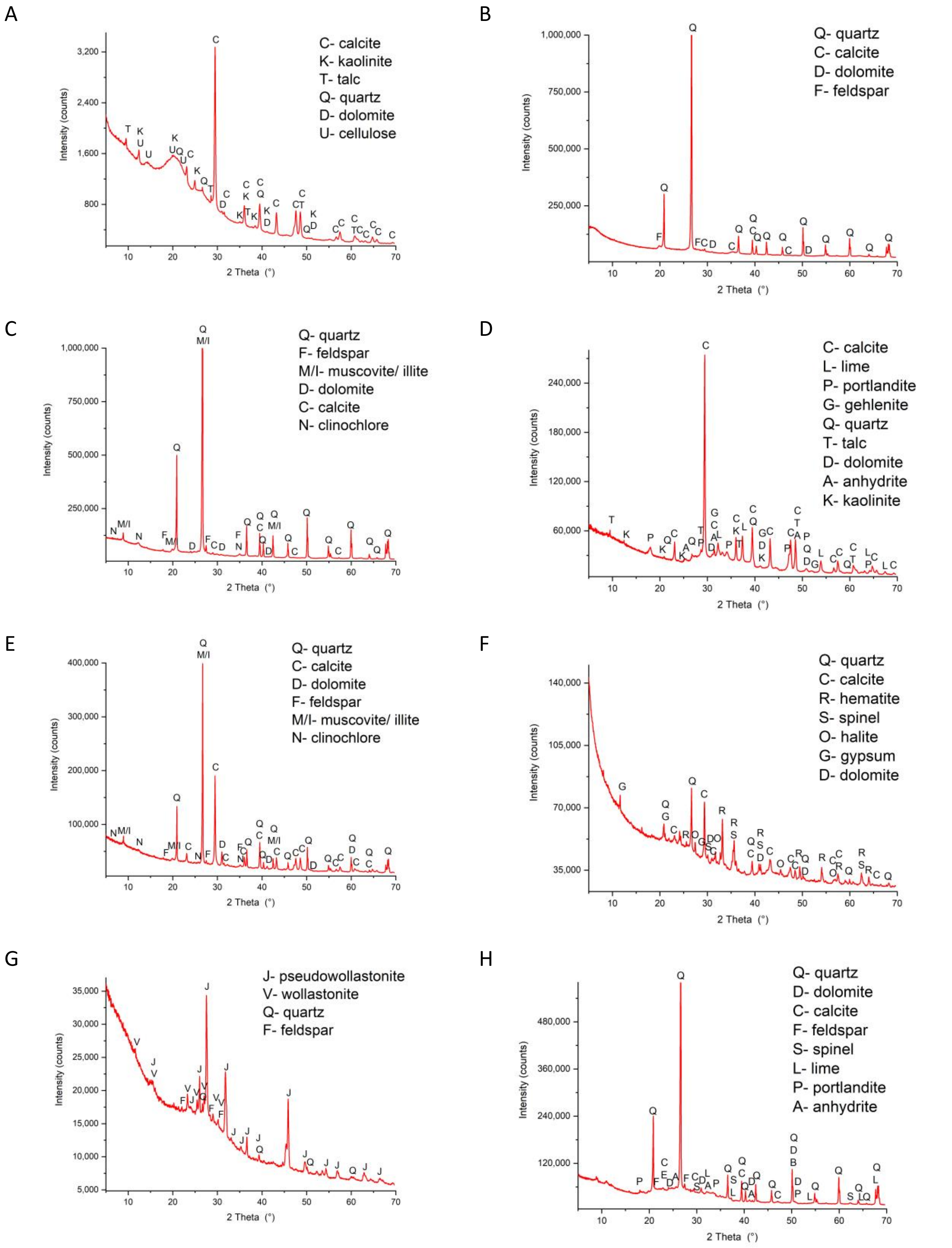

Figure S5. XRD patterns of raw materials (A) Paper mill sludge, (B) Foundry sand, (C) Mine waste, (D) Paper ash, (E) Digestate, (F) Bottom ash from incineration of waste, (G) Foundry slag and (H) Coal ash.
