# Supplementary material for: Environmental Acceptability of Geotechnical Composites from Recycled Materials: Comparative Study of Laboratory and Field Investigations
Source: Int J Environ Res Public Health. 2023 Jan 21;20(3):2014. doi: 10.3390/ijerph20032014 (PMC9915455; doi:10.3390/ijerph20032014)
Supplement: Supplementary file 1 [file ijerph-20-02014-s001.zip › Figure s7.pdf]

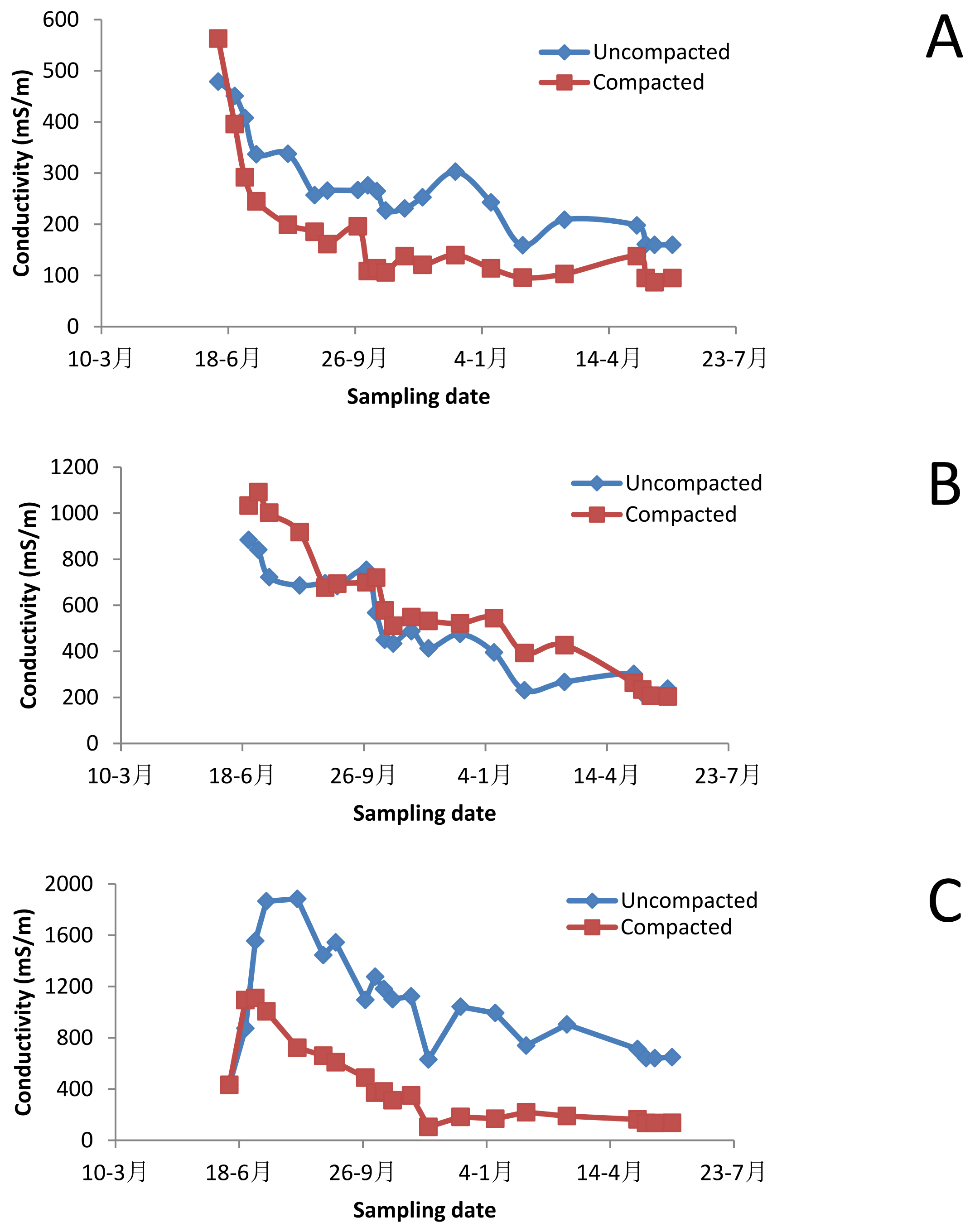

Figure S7 Conductivity values (mS/m) of percolates from (A) Composite 1, (B) Composite 2 and (C) Composite 3 as function of different sampling period. Results represent values obtained from samplings in a period from June 2020 to June 2021.
