# Supplementary material for: Environmental Acceptability of Geotechnical Composites from Recycled Materials: Comparative Study of Laboratory and Field Investigations
Source: Int J Environ Res Public Health. 2023 Jan 21;20(3):2014. doi: 10.3390/ijerph20032014 (PMC9915455; doi:10.3390/ijerph20032014)
Supplement: Supplementary file 1 [file ijerph-20-02014-s001.zip › Tables Supplementary.pdf]

Table S1
Basic information about the input raw materials used in the experiments.

**Type of input raw material**	**Abbreviation used in the text**	**European waste code***	**Source of a raw material**	**Contact information of the source company**
Paper-mill sludge	PMS	03 03 10	Paper mill for production of label and packaging paper	Brigl & Bergmeister GmbH –Vevče, Papirniška pot 25, 1261 Ljubljana, Slovenia
Paper ash	PA	10 01 01	Paper mill for production of newsprint and packaging paper	VIPAP Videm Krško d.d., Tovarniška ulica 18, Krško, 8270 Krško
Coal fly ash	CA	10 01 02		
Solid-waste incineration bottom ash	SWIA	19 01 12	Chemical products factory and incineration of waste	Albaugh TKI d.o.o. (Formerly PINUS TKI), Grajski trg 21, Rače, 2327 Rače, Slovenia
Foundry slag	FSL	10 09 03	Foundry for production and mechanical processing of metal castings	Livar d.d., Ljubljanska cesta 43, 1295 Ivančna Gorica, Slovenia
Foundry sand	FS	10 09 08		
Digestate from the mechanical-biological anaerobic treatment of municipal solid waste	DI	19 06 04	Public utility company – Regional Waste Management Centre – RCERO	JP VOKA SNAGA d.o.o., Vodovodna cesta 90, 1000 Ljubljana, Slovenia
Mining waste	MW	01 04 09	Mining company for the production and processing of silica sands	Termit d.d., Drtija 51, 1251 Moravče, Slovenia

* European Union's List of Wastes (European Commission, 2000)

Table S2
Maximum reference dry density and optimal moisture content of geotechnical composites

Parameter	Composite 1	Composite 2	Composite 3
Maximum reference dry density (Mg/m^3)	1.83	1.12	1.48
Optimal water content (%)	13.8	34.0	22.6

Table S3a

Precipitation, volume of collected lysimetric water, concentrations of PTEs and anions (dry mass basis), pH and conductivity in lysimetic water from uncompacted Composite 1.

Parameter	Sampling period (2020)														Sampling period (2021)							Average result	Standard deviation
	**4.6.-10.6.**	**10.6.-23.6.**	**23.6.-1.7.**	**1.7.-10.7.**	**10.7.-4.8.**	**4.8.-25.8.**	**25.8.-4.9.**	**4.9.-28.9.**	**28.9.-6.10.**	**6.10.-13.10.**	**13.10.-20.10.**	**20.10.-4.11.**	**4.11.-18.11.**	**18.11.-14.12.**	**14.12.-11.1.**	**11.1.-5.2.**	**5.2.-10.3.**	**10.3.-6.5.**	**6.5.-13.5.**	**13.5.-20.5.**	**20.5.-3.6.**		
Precipitation (mm)	76	86	48	63	182	66	51	120	79	61	28	22	44	45	165	66	45	150	47	75	77	76	43
Volume of collected water (L)	470	650	310	460	1100	500	310	780	600	400	100	160	250	430	1200	520	625	890	400	460	550	526	230
As (mg/kg)	0,0006	0,0006	0,0003	0,0004	0,0010	0,0003	0,0002	0,0005	0,0003			0,00009	0,0001	0,0002		0,0001	0,0002	0,0002	0,00009	0,00008	0,0001	0,0003	0,0002
Ba (mg/kg)	0,0052	0,0033	0,0012	0,0025	0,0053	0,0031	0,0018	0,0058	0,0049			0,0005	0,0013	0,0022		0,0018	0,0024	0,0015	0,0004	0,0025	0,0025	0,0027	0,0017
Cd (mg/kg)	0,000007	0,00001	0,000005	0,000007	0,00002	0,000007	0,000005	0,00001	0,000009			0,000002	0,000004	0,000006		0,000008	0,000009	0,00001	0,000006	0,000007	0,00001	0,000008	0,000003
Cr total (mg/kg)	0,00002	0,00001	0,000007	0,00001	0,00004	0,000007	0,000005	0,00001	0,00001			0,000005	0,000006	0,000010		0,000008	0,000009	0,00002	0,000009	0,000007	0,0000	0,00001	0,000009
Cu (mg/kg)	0,00033	0,0004	0,0002	0,0003	0,0005	0,0002	0,00006	0,0002	0,0002			0,00007	0,00009	0,0002		0,00009	0,0002	0,0002	0,00008	0,0001	0,00	0,0002	0,0001
Hg (mg/kg)	0,000003	0,000005	0,000002	0,000003	0,000008	0,000004	0,000002	0,000006	0,000004			0,000001	0,000002	0,000003		0,000004	0,000005	0,000007	0,000003	0,000003	0,000004	0,000004	0,000002
Mo (mg/kg)	0,0019	0,0027	0,0015	0,0019	0,0047	0,0024	0,0013	0,0034	0,0024	Technical problem	Technical problem	0,0007	0,0008	0,0016	Technical problem	0,0012	0,0017	0,0025	0,0007	0,0007	0,001	0,0018	0,0011
Ni (mg/kg)	0,0017	0,002	0,0007	0,0011	0,0027	0,0009	0,0005	0,0013	0,0012			0,0003	0,0004	0,0008		0,0005	0,0007	0,0007	0,0004	0,0005	0,0006	0,0009	0,0006
Pb (mg/kg)	0,00002	0,00002	0,00001	0,00002	0,00004	0,00002	0,00001	0,00003	0,00002			0,000006	0,000009	0,00002		0,00002	0,00002	0,00003	0,00001	0,00002	0,00002	0,00002	0,000009
Sb (mg/kg)	0,00005	0,00007	0,00003	0,00004	0,0001	0,00003	0,00002	0,00006	0,00005			0,00001	0,00001	0,00003		0,00003	0,00005	0,00007	0,00003	0,00003	0,0000	0,00004	0,00002
Se (mg/kg)	0,00023	0,0002	0,00006	0,0001	0,0003	0,00008	0,00003	0,0001	0,00006			0,00002	0,00001	0,00004		0,00003	0,00004	0,00010	0,00001	0,00002	0,0000	0,00008	0,00008
Zn (mg/kg)	0,023	0,012	0,0006	0,012	0,019	0,0019	0,00004	0,012	0,018			0,0003	0,0061	0,0077		0,0097	0,0074	0,0012	0,0002	0,019	0,0094	0,0088	0,0074
Chloride (mg/kg)	7,3	8,2	12,7	4,4	11,4	5,2	2,3	4,1	3,8			1,1	6,0	1,3		0,77	1,5	2,3	0,51	0,44	0,7	4,1	3,7
Fluoride (mg/kg)	0,043	0,049	0,030	0,044	0,10	0,046	0,031	0,08	0,05			0,01	0,02	0,03		0,036	0,047	0,058	0,031	0,026	0,042	0,043	0,021
Sulfate (mg/kg)	48,6	54,1	22,1	28,4	71,9	25,8	12,9	38,0	25,6			6,9	10,9	12,1		10,2	17,9	29,8	8,6	7,9	8,6	24,5	18,4
phenol index																							
pH	7,2	7,5	7,8	8,0	7,6	7,6	7,8	7,7	7,3	7,8	8,9	8,1	7,9	7,7	8,4	7,5	8,0	8,0	7,7	7,0	7,3	7,8	0,42
Conductivity (mS/m)	479	451	408	337	338	257	266	267	276	265	227	231	253	303	243	159	209	198	161	160	160	269	92
L/S	0,035	0,048	0,023	0,034	0,082	0,037	0,023	0,058	0,045	0,030	0,007	0,012	0,019	0,032	0,089	0,039	0,046	0,066	0,030	0,034	0,041		
L/S (cumulative)	0,035	0,083	0,106	0,141	0,222	0,259	0,283	0,341	0,385	0,415	0,422	0,434	0,453	0,485	0,574	0,613	0,659	0,725	0,755	0,789	0,830		

Table S3a (continued)

Precipitation, volume of collected lysimetric water, concentrations of PTEs and anions (dry mass basis), pH and conductivity in lysimetic water from compacted Composite 1.

Parameter	Sampling period (2020)														Sampling period (2021)							Average result	Standard deviation
	**4.6.-10.6.**	**10.6.-23.6.**	**23.6.-1.7.**	**1.7.-10.7.**	**10.7.-4.8.**	**4.8.-25.8.**	**25.8.-4.9.**	**4.9.-28.9.**	**28.9.-6.10.**	**6.10.-13.10.**	**13.10.-20.10.**	**20.10.-4.11.**	**4.11.-18.11.**	**18.11.-14.12.**	**14.12.-11.1.**	**11.1.-5.2.**	**5.2.-10.3.**	**10.3.-6.5.**	**6.5.-13.5.**	**13.5.-20.5.**	**20.5.-3.6.**		
Precipitation (mm)	76	86	48	63	182	66	51	120	79	61	28	22	44	45	165	66	45	150	47	75	77	76	43
Volume of collected water (L)	360	550	220	340	770	390	280	610	570	470	270	100	170	410	700	520	570	690	300	350	350	415	176
As (mg/kg)	0,00004	0,00005	0,00002	0,00003	0,00005	0,00002	0,00001	0,00004	0,00002	0,00002	0,000007	0,00000	0,000005	0,00002		0,00001	0,00002	0,00002	0,00001	0,00001		0,00009	0,0003
Ba (mg/kg)	0,0019	0,0008	0,0006	0,0013	0,0031	0,0018	0,0012	0,0025	0,0025	0,002	0,000600	0,0004	0,0005	0,0013		0,0010	0,0015	0,0009	0,0002	0,001		0,0014	0,0009
Cd (mg/kg)	0,000004	0,00001	0,000002	0,000004	0,00001	0,000004	0,000003	0,00001	0,000006	0,000005	0,000003	0,000001	0,000002	0,000004		0,000006	0,000006	0,00001	0,000003	0,000004		0,000007	0,000009
Cr total (mg/kg)	0,00003	0,00002	0,000007	0,00002	0,00003	0,00001	0,000006	0,00002	0,00002	0,00001	0,000003	0,000003	0,000006	0,000013		0,000006	0,00002	0,00003	0,000006	0,000010		0,0002	0,0008
Cu (mg/kg)	0,0002	0,0002	0,00008	0,0001	0,0002	0,00008	0,00005	0,0001	0,00007	0,00006	0,000028	0,00001	0,00002	0,00003		0,00004	0,00004	0,00006	0,00002	0,0000		0,009	0,038
Hg (mg/kg)	0,000002	0,000003	0,000001	0,000002	0,000004	0,000002	0,000002	0,000003	0,000003	0,000003	0,000001	0,000001	0,000001	0,000002		0,000003	0,000003	0,000004	0,000002	0,000010		0,000003	0,000002
Mo (mg/kg)	0,0007	0,0012	0,0005	0,0008	0,0014	0,0008	0,00046	0,0014	0,0009	0,0007	0,0004	0,0002	0,0002	0,0007		0,0009	0,0009	0,0013	0,0004	0,0003		0,0026	0,0079
Ni (mg/kg)	0,0003	0,0004	0,0001	0,0002	0,0004	0,0002	0,0001	0,0003	0,0002	0,0002	0,00008	0,00003	0,00004	0,0001	Technical problem	0,00009	0,00010	0,0001	0,00004	0,00005	Technical problem	0,0009	0,0030
Pb (mg/kg)	0,00001	0,00002	0,00001	0,00001	0,00002	0,00001	0,00001	0,00002	0,00002	0,00001	0,000007	0,000003	0,000005	0,00001		0,00001	0,00002	0,00002	0,00001	0,00001		0,000012	0,000005
Sb (mg/kg)	0,00002	0,00004	0,00001	0,00002	0,00005	0,00002	0,00001	0,00002	0,00002	0,00001	0,000006	0,000003	0,000003	0,00001		0,00001	0,00001	0,00002	0,00001	0,00001		0,00005	0,0001
Se (mg/kg)	0,00004	0,00003	0,00001	0,00001	0,00004	0,00001	0,00001	0,00001	0,00001	0,000008	0,000004	0,000002	0,000002	0,00001		0,00001	0,00001	0,00002	0,00000	0,00001		0,00003	0,00006
Zn (mg/kg)	0,0001	0,00002	0,00001	0,00001	0,00002	0,0000	0,00001	0,00002	0,00006	0,00005	0,000007	0,000003	0,00002	0,00001		0,00001	0,00002	0,00002	0,000008	0,00006		0,00004	0,00007
Chloride (mg/kg)	14,0	21,9	2,3	9,5	14,3	11,0	4,9	16,1	6,8	3,6	2,9	1,4	2,2	5,6		5,0	5,0	10,0	2,8	2,4		8,5	6,1
Fluoride (mg/kg)	0,022	0,036	0,016	0,022	0,05	0,029	0,016	0,04	0,03	0,036	0,017	0,01	0,01	0,02		0,029	0,034	0,037	0,015	0,016		0,03	0,01
Sulfate (mg/kg)	18,3	18,6	3,4	7,3	8,0	4,2	2,3	5,6	3,0	2,59	1,25	0,7	1,6	2,6		1,6	2,0	5,3	1,6	1,4		5,8	5,9
phenol index					0,0002					0,0001			0,00005							0,00010		0,00012	0,00008
pH	7,5	7,9	8,1	8,0	8,1	8,1	8,0	8,0	7,5	7,8	8,1	7,9	8,0	8,1	8,3	8,0	8,3	8,2	8,1	7,9	8,1	8,0	0,2
Conductivity (mS/m)	563	396	292	245	199,4	185,6	161,3	196,1	108,6	114	106,1	138	120,8	140	114	96	103	138	95	87	95	176	117
L/S	0,020	0,030	0,012	0,019	0,042	0,021	0,015	0,033	0,031	0,026	0,015	0,005	0,009	0,022	0,038	0,028	0,031	0,038	0,016	0,019	0,019		
L/S (cumulative)	0,020	0,050	0,062	0,080	0,122	0,144	0,159	0,192	0,223	0,249	0,264	0,269	0,278	0,301	0,339	0,367	0,398	0,436	0,453	0,472	0,491		

Table S3b

Precipitation, volume of collected lysimetric water, concentrations of PTEs and anions (dry mass basis), pH and conductivity in lysimetic water from uncompacted Composite 2.

Parameter	Sampling period (2020)													Sampling period (2021)								
	**10.6.-23.6.**	**23.6.-1.7.**	**1.7.-10.7.**	**10.7.-4.8.**	**4.8.-25.8.**	**25.8.-4.9.**	**4.9.-28.9.**	**28.9.-6.10.**	**6.10.-13.10.**	**13.10.-20.10.**	**20.10.-4.11.**	**4.11.-18.11.**	**18.11.-14.12.**	**14.12.-11.1.**	**11.1.-5.2.**	**5.2.-10.3.**	**10.3.-6.5.**	**6.5.-13.5.**	**13.5.-20.5.**	**20.5.-3.6.**	Average result	Standard deviation
Precipitation (mm)	86	48	63	182	66	51	120	79	61	28	22	44	45	165	66	45	150	47	75	77	76	45
Volume of collected water (L)	340	300	455	1100	490	325	760	640	410	225	100	205	430	1300	580	640	900	420	350	660	532	303
As (mg/kg)	0,0033	0,0029	0,0043	0,011	0,0034	0,0020	0,0051	0,0045	0,0024	0,0011		0,0013	0,0025		0,002200	0,0023		0,0012	0,0012	0,0021	0,0031	0,0024
Ba (mg/kg)	0,0082	0,0036	0,0055	0,042	0,0075	0,0004	0,0002	0,0029	0,0037	0,0017		0,0020	0,00009		0,000900	0,0015		0,0005	0,0014	0,0021	0,0049	0,0099
Cd (mg/kg)	0,00003	0,00003	0,00005	0,0001	0,00006	0,00006	0,0001	0,0001	0,00005	0,00004		0,00003	0,00004		0,00006	0,00006		0,00003	0,00003	0,00005	0,00005	0,00002
Cr total (mg/kg)	0,0027	0,0023	0,0037	0,0086	0,0036	0,0026	0,0051	0,0052	0,0033	0,0015		0,0019	0,0034		0,0034	0,0038		0,0037	0,0021	0,0044	0,0036	0,0017
Cu (mg/kg)	0,95	0,9	1,2	4,1	1,0	0,57	1,2	1,2	0,58	0,018		0,26	0,57		0,34	0,35		0,20	0,24	0,43	0,83	0,93
Hg (mg/kg)	0,000004	0,000003	0,000005	0,00001	0,000005	0,000004	0,000008	0,000007	0,000004	0,000002		0,000002	0,000005		0,00002	0,00001		0,00002	0,000008	0,00001	0,000008	0,000006
Mo (mg/kg)	0,03	0,028	0,042	0,11	0,053	0,034	0,077	0,071	0,041	0,021	Technical problem	0,020	0,041	Technical problem	0,039	0,038	Technical problem	0,011	0,019	0,052	0,043	0,025
Ni (mg/kg)	0,071	0,066	0,089	0,26	0,071	0,043	0,083	0,084	0,04	0,0037		0,016	0,038		0,023	0,024		0,014	0,013	0,025	0,057	0,059
Pb (mg/kg)	0,00001	0,00001	0,00002	0,00006	0,00007	0,00006	0,00004	0,00004	0,00007	0,0001		0,00002	0,00002		0,00004	0,00004		0,00002	0,00001	0,00002	0,00004	0,00003
Sb (mg/kg)	0,00007	0,0001	0,0002	0,0004	0,0003	0,0002	0,0007	0,0005	0,0004	0,0002		0,0002	0,0004		0,0004	0,0005		0,0004	0,0001	0,0002	0,0003	0,0002
Se (mg/kg)	0,0004	0,0004	0,0012	0,011	0,0016	0,0006	0,0009	0,0008	0,00001	0,000007		0,0002	0,00001		0,0003	0,0004		0,0002	0,0002	0,0003	0,0011	0,0026
Zn (mg/kg)	0,0005	0,0001	0,0002	0,0007	0,0004	0,0005	0,00004	0,0008	0,0004	0,0002		0,0008	0,00002		0,0005	0,0004		0,0003	0,00007	0,0026	0,0005	0,0006
Chloride (mg/kg)	27,1	26,2	33,3	85,3	35,3	36,6	65,6	41,9	3,1	12,7		11,2	22,8		17,7	23,4		12,4	15,7	25,9	29,2	20,5
Fluoride (mg/kg)	0,04	0,04	0,06	0,11	0,06	0,04	0,08	0,04	0,04	0,02		0,010	0,038		0,06	0,06		0,03	0,03	0,06	0,049	0,023
Sulfate (mg/kg)	5,38	5,93	9,04	14,4	10,2	7,5	23,7	10,8	6,5	3,2		3,4	11,7		4,9	10,4		5,5	1,8	3,3	8,1	5,3
phenol index																						
pH	11,0	10,8	12,7	10,6	11,8	10,2	9,6	10,2	10,0	10,0	9,6	9,4	9,2	9,2	9,2	8,9	8,5	8,7	10,3	7,7	9,9	1,2
Conductivity (mS/m)	884	841	722	686	696	683	754	568	450	434	487	413	475	395	231	267	302	219	212	238	498	219
L/S	0,037	0,033	0,050	0,120	0,053	0,035	0,083	0,070	0,045	0,025	0,011	0,022	0,047	0,142	0,063	0,070	0,098	0,046	0,038	0,072		
L/S (cumulative)	0,037	0,070	0,12	0,24	0,29	0,33	0,41	0,48	0,53	0,55	0,56	0,58	0,63	0,77	0,84	0,91	1,00	1,05	1,09	1,16		

Table S3b (continued)

Precipitation, volume of collected lysimetric water, concentrations of PTEs and anions (dry mass basis), pH and conductivity in lysimetic water from compacted Composite 2.

Parameter	Sampling period (2020)													Sampling period (2021)								
	**10.6.-23.6.**	**23.6.-1.7.**	**1.7.-10.7.**	**10.7.-4.8.**	**4.8.-25.8.**	**25.8.-4.9.**	**4.9.-28.9.**	**28.9.-6.10.**	**6.10.-13.10.**	**13.10.-20.10.**	**20.10.-4.11.**	**4.11.-18.11.**	**18.11.-14.12.**	**14.12.-11.1.**	**11.1.-5.2.**	**5.2.-10.3.**	**10.3.-6.5.**	**6.5.-13.5.**	**13.5.-20.5.**	**20.5.-3.6.**	Average result	Standard deviation
Precipitation (mm)	86	48	63	182	66	51	120	79	61	28	22	44	45	165	66	45	150	47	75	77	76	45
Volume of collected water (L)	320	310	475	1100	520	340	740	660	500	310	220	250	480	1000	640	565	950	510	480	680	553	248
As (mg/kg)	0,0022	0,0027	0,0039	0,0071	0,0033	0,0019	0,0040	0,0043	0,0027	0,0015	0,0012	0,0014	0,0026		0,0022	0,0022		0,0015	0,0008	0,0012	0,0027	0,0015
Ba (mg/kg)	0,0083	0,0086	0,015	0,0019	0,019	0,0015	0,0001	0,018	0,015	0,0073	0,0012	0,013	0,0036		0,0078	0,0098		0,0008	0,0015	0,0035	0,0080	0,0063
Cd (mg/kg)	0,00002	0,00002	0,00004	0,00007	0,00005	0,00003	0,00008	0,00008	0,00005	0,00004	0,00002	0,00002	0,00001		0,00005	0,00004		0,00003	0,00003	0,00004	0,00004	0,00002
Cr total (mg/kg)	0,0014	0,0016	0,0024	0,0082	0,0023	0,0015	0,0028	0,0030	0,0023	0,0012	0,0007	0,0008	0,0015		0,0015	0,0015		0,0020	0,0031	0,0034	0,0024	0,0017
Cu (mg/kg)	0,76	1,0	1,3	2,0	1,3	0,74	1,4	1,6	0,92	0,028	0,38	0,40	0,72		0,41	0,44		0,31	0,12	0,16	0,81	0,57
Hg (mg/kg)	0,000003	0,000003	0,000004	0,00001	0,0003	0,000006	0,000006	0,000006	0,000004	0,000003	0,000002	0,000002	0,000004		0,000006	0,00001		0,00002	0,000008	0,000006	0,000022	0,000064
Mo (mg/kg)	0,018	0,023	0,032	0,081	0,043	0,027	0,053	0,058	0,037	0,022	0,017	0,018	0,028		0,030	0,026		0,026	0,021	0,034	0,034	0,017
Ni (mg/kg)	0,050	0,067	0,087	0,14	0,080	0,047	0,084	0,10	0,056	0,0051	0,023	0,023	0,043	Technical problem	0,023	0,024	Technical problem	0,018	0,0083	0,013	0,051	0,038
Pb (mg/kg)	0,000008	0,000008	0,00002	0,00005	0,00002	0,0002	0,00003	0,00003	0,00002	0,0002	0,00002	0,00001	0,00002		0,00003	0,0001		0,00002	0,00003	0,00002	0,00005	0,00006
Sb (mg/kg)	0,00003	0,00005	0,0001	0,0004	0,0002	0,0001	0,0003	0,0002	0,0001	0,00007	0,00006	0,00007	0,00005		0,00006	0,00007		0,00010	0,0004	0,0006	0,0002	0,0002
Se (mg/kg)	0,0003	0,0003	0,0010	0,0095	0,0013	0,0005	0,0006	0,0007	0,00001	0,000008	0,000006	0,0002	0,00001		0,00024	0,0003		0,0002	0,0003	0,0003	0,0009	0,0022
Zn (mg/kg)	0,0013	0,0002	0,0009	0,0070	0,0002	0,0005	0,00003	0,0008	0,0015	0,0015	0,0006	0,0012	0,00027		0,0011	0,0009		0,0001	0,0002	0,0003	0,0011	0,0016
Chloride (mg/kg)	14,7	19,6	32,2	71,6	22,1	24,8	57,2	32,1	3,5	15,3	9,1	8,7	18,5		16,1	17,2		15,3	12,1	17,1	23,4	17,2
Fluoride (mg/kg)	0,04	0,04	0,05	0,11	0,07	0,04	0,04	0,05	0,05	0,03	0,017	0,02	0,04		0,062	0,055		0,043	0,032	0,034	0,048	0,020
Sulfate (mg/kg)	3,3	4,4	6,8	10,5	4,7	4,0	12,1	6,9	4,3	2,7	1,9	5,4	2,8		1,3	1,6		1,5	9,2	16,6	5,8	4,2
phenol index																						
pH	11,2	11,1	13,2	12,3	10,2	10,9	10	11,7	11,7	12	10,8	9,9	11,3	11,9	12	11,8	9,7	10,9	7,8	9,5	11,0	1,2
Conductivity (mS/m)	1033	1092	1002	918	677	694	700	720	578	511	549	532	521	544	393	427	263	234	207	204	590	270
L/S	0,028	0,027	0,041	0,095	0,045	0,030	0,064	0,057	0,043	0,027	0,019	0,022	0,042	0,087	0,056	0,049	0,082	0,044	0,042	0,059		
L/S (cumulative)	0,028	0,055	0,096	0,19	0,24	0,27	0,33	0,39	0,43	0,46	0,48	0,50	0,54	0,63	0,68	0,73	0,81	0,86	0,90	0,96		

Table S3c

Precipitation, volume of collected lysimetric water, concentrations of PTEs and anions (dry mass basis), pH and conductivity in lysimetic water from uncompacted Composite 3.

Parameter	Sampling period (2020)														Sampling period (2021)								
	**10.6.-17.6.**	**17.6.-23.6.**	**23.6.-1.7.**	**1.7.-10.7.**	**10.7.-4.8.**	**4.8.-25.8.**	**25.8.-4.9.**	**4.9.-28.9.**	**28.9.-6.10.**	**6.10.-13.10.**	**13.10.-20.10.**	**20.10.-4.11.**	**4.11.-18.11.**	**18.11.-14.12.**	**14.12.-11.1.**	**11.1.-5.2.**	**5.2.-10.3.**	**10.3.-6.5.**	**6.5.-13.5.**	**13.5.-20.5.**	**20.5.-3.6.**	Average results	Standard deviation
Precipitation (mm)	40	86	48	63	182	66	51	120	79	61	28	22	44	45	165	66	45	150	47	75	77	74	44
Volume of collected water (L)	5,5	100	305	445	940	460	330	550	520	400	280	190	100	400	800	570	575	600	290	240	390	404	228
As (mg/kg)	0,00002	0,0008	0,0052	0,0046	0,0025	0,0016	0,0006	0,0009	0,0008	0,0004	0,0001	0,00024	0,00009	0,0002		0,00007	0,00008	0,0002	0,00003	0,00003	0,00003	0,0007	0,0012
Ba (mg/kg)	0,00007	0,0021	0,0045	0,032	0,039	0,0089	0,032	0,026	0,042	0,033	0,023	0,0069	0,0040	0,011		0,053	0,073	0,0003	0,0019	0,029	0,015	0,024	0,020
Cd (mg/kg)	0,0000001	0,0000	0,0000	0,00003	0,00001	0,00004	0,000005	0,00004	0,00005	0,00004	0,00002	0,00002	0,000004	0,00003		0,00003	0,00003	0,00003	0,00001	0,000009	0,00001	0,00002	0,00001
Cr total (mg/kg)	0,00002	0,0007	0,0039	0,0073	0,0034	0,0048	0,00081	0,0025	0,0021	0,0011	0,0005	0,0004	0,0001	0,0005		0,0004	0,0004	0,0004	0,0002	0,0002	0,0004	0,0014	0,0020
Cu (mg/kg)	0,00002	0,0004	0,0012	0,0010	0,0010	0,0009	0,0002	0,0005	0,0004	0,0002	0,00003	0,0002	0,00005	0,0001		0,00009	0,0001	0,0002	0,00005	0,00005	0,00009	0,0003	0,0003
Hg (mg/kg)	0,0000001	0,0000	0,0000	0,00002	0,00004	0,00003	0,000002	0,00004	0,000004	0,000003	0,000004	0,000008	0,000001	0,000006		0,000004	0,000004	0,00004	0,000009	0,000002	0,000003	0,00001	0,00001
Mo (mg/kg)	0,00009	0,0030	0,0141	0,024	0,013	0,035	0,0037	0,030	0,040	0,027	0,016	0,013	0,0035	0,020	Technical problems	0,017	0,012	0,026	0,0091	0,0070	0,015	0,017	0,011
Ni (mg/kg)	0,000005	0,0001	0,0003	0,0002	0,00009	0,0002	0,00006	0,0001	0,00009	0,00004	0,000007	0,00003	0,000007	0,00003		0,00001	0,00003	0,00001	0,00001	0,00001	0,00002	0,00005	0,00006
Pb (mg/kg)	0,0000002	0,0000	0,0000	0,00002	0,00004	0,00002	0,00001	0,00002	0,00002	0,00001	0,00001	0,000007	0,000004	0,00001		0,00002	0,00002	0,00002	0,00001	0,00001	0,00001	0,000015	0,000008
Sb (mg/kg)	0,000001	0,0000	0,0001	0,00007	0,00008	0,00007	0,00002	0,00005	0,00004	0,00002	0,000004	0,00002	0,000004	0,00001		0,000004	0,000004	0,00003	0,000002	0,000007	0,00001	0,00003	0,00003
Se (mg/kg)	0,000003	0,0001	0,0004	0,0009	0,0013	0,0008	0,0002	0,0008	0,0007	0,0006	0,0003	0,0002	0,00007	0,0004		0,0003	0,0004	0,0008	0,0002	0,0001	0,0002	0,0005	0,0004
Zn (mg/kg)	0,000001	0,0000	0,0000	0,00006	0,00004	0,0003	0,00002	0,00002	0,0010	0,0009	0,00001	0,00009	0,0006	0,0011		0,00008	0,0004	0,00002	0,00002	0,000009	0,0005	0,0003	0,0004
Chloride (mg/kg)	0,03	1,1183	6,1393	10,0	23,8	12,3	9,3	11,7	8,7	6,0	4,6	3,5	1,0	3,1		5,1	6,0	8,1	2,7	2,3	4,2	6,8	5,5
Fluoride (mg/kg)	0,00	0,0101	0,0314	0,05	0,10	0,05	0,03	0,06	0,05	0,05	0,03	0,01	0,01	0,03		0,03	0,04	0,04	0,02	0,02	0,03	0,035	0,022
Sulfate (mg/kg)	0,01	0,1491	0,5230	0,66	2,6	1,03	0,49	0,82	0,93	0,60	0,42	0,28	0,15	0,60		0,85	0,86	0,89	0,43	0,36	0,58	0,70	0,55
phenol index																							
pH	12,2	11,5	11,7	13,9	13,1	13,6	12,8	12,7	12,7	12,9	13,1	12,6	12,3	12,6	12,6	12,7	12,4	12,8	12,6	12,6	12,6	12,7	0,5
Conductivity (mS/m)	430	874	1556	1865	1884	1445	1544	1095	1276	1181	1101	1123	630	1042	993	739	904	712	640	640	649	1063	413
L/S	0,0004	0,007	0,023	0,033	0,070	0,034	0,025	0,041	0,039	0,030	0,021	0,014	0,007	0,030	0,060	0,043	0,043	0,045	0,022	0,018	0,029		
L/S (cumulative)	0,0004	0,008	0,031	0,064	0,13	0,17	0,19	0,23	0,27	0,30	0,32	0,34	0,34	0,37	0,43	0,48	0,52	0,56	0,59	0,60	0,63		

Table S3c (continued)

Precipitation, volume of collected lysimetric water, concentrations of PTEs and anions (dry mass basis), pH and conductivity in lysimetic water from compacted Composite 3.

Parameter	Sampling period (2020)														Sampling period (2021)								
	**10.6.-17.6.**	**17.6.-23.6.**	**23.6.-1.7.**	**1.7.-10.7.**	**10.7.-4.8.**	**4.8.-25.8.**	**25.8.-4.9.**	**4.9.-28.9.**	**28.9.-6.10.**	**6.10.-13.10.**	**13.10.-20.10.**	**20.10.-4.11.**	**4.11.-18.11.**	**18.11.-14.12.**	**14.12.-11.1.**	**11.1.-5.2.**	**5.2.-10.3.**	**10.3.-6.5.**	**6.5.-13.5.**	**13.5.-20.5.**	**20.5.-3.6.**	Average results	Standard deviation
Precipitation (mm)	40	86	48	63	182	66	51	120	79	61	28	22	44	45	165	66	45	150	47	75	77	74	44
Volume of collected water (L)	60	100	60	75	850	325	280	430	520	380	230	125	180	270	600	555	575	555	280	325	420	343	213
As (mg/kg)	0,0005			0,0001	0,0048	0,0007	0,0007	0,0008	0,0001	0,00006	0,00003	0,00005	0,00006	0,00004		0,00001	0,00003	0,00006	0,000005	0,00002	0,000005	0,0004	0,0011
Ba (mg/kg)	0,0011			0,0057	0,048	0,0084	0,018	0,0090	0,032	0,031	0,014	0,0015	0,0036	0,0031		0,031	0,018	0,0004	0,0026	0,018	0,011	0,014	0,014
Cd (mg/kg)	0,000001			0,000001	0,00005	0,000006	0,00002	0,000005	0,000007	0,000005	0,000003	0,000002	0,000002	0,000003		0,000007	0,000007	0,000007	0,000004	0,000004	0,000005	0,00001	0,00001
Cr total (mg/kg)	0,0001			0,0002	0,011	0,0010	0,0018	0,0010	0,0005	0,0003	0,0001	0,0001	0,0001	0,0001		0,0003	0,0003	0,0003	0,0001	0,0002	0,0002	0,0010	0,0025
Cu (mg/kg)	0,0002			0,00008	0,0014	0,0004	0,0003	0,0004	0,0001	0,00004	0,0001	0,00003	0,00003	0,00001		0,00002	0,00006	0,00005	0,00001	0,00002	0,00002	0,0002	0,0003
Hg (mg/kg)	0,000002			0,000001	0,00004	0,00002	0,000004	0,00002	0,00001	0,000002	0,000001	0,000004	0,000002	0,000002		0,000004	0,000004	0,000004	0,000002	0,000002	0,000003	0,00001	0,00001
Mo (mg/kg)	0,0006	Technical problems	Technical problems	0,0009	0,043	0,0059	0,017	0,0043	0,0031	0,0021	0,0011	0,0008	0,0003	0,0006	Technical problems	0,0011	0,0012	0,0019	0,0007	0,0007	0,0013	0,005	0,010
Ni (mg/kg)	0,00008			0,00002	0,0004	0,0001	0,00007	0,0001	0,00003	0,00001	0,00003	0,000007	0,000007	0,000009		0,000007	0,00001	0,000007	0,000004	0,000006	0,000005	0,00005	0,00009
Pb (mg/kg)	0,00001			0,000002	0,00003	0,00001	0,000009	0,00001	0,00002	0,00001	0,000007	0,000004	0,000006	0,000009		0,00002	0,00002	0,00002	0,000009	0,00001	0,00001	0,000012	0,000006
Sb (mg/kg)	0,000006			0,000005	0,0001	0,00004	0,00003	0,00003	0,00002	0,00001	0,000006	0,000005	0,000006	0,000007		0,000007	0,00001	0,00001	0,000007	0,00001	0,00001	0,00002	0,00003
Se (mg/kg)	0,00004			0,00006	0,0019	0,0002	0,0004	0,0002	0,00009	0,00006	0,00005	0,00003	0,00003	0,00004		0,00001	0,00005	0,0001	0,00002	0,00002	0,00003	0,0002	0,0004
Zn (mg/kg)	0,00006			0,0003	0,0004	0,00006	0,00002	0,00001	0,0013	0,0011	0,000007	0,000004	0,0003	0,0004		0,00006	0,0003	0,00002	0,00001	0,00001	0,0010	0,0003	0,0004
Chloride (mg/kg)	0,26			0,52	5,4	2,5	1,7	3,0	2,0	13,2	0,65	0,47	0,17	0,26		0,88	1,2	1,3	0,39	0,57	0,66	1,9	3,1
Fluoride (mg/kg)	0,01			0,01	0,07	0,03	0,02	0,03	0,02	0,02	0,01	0,01	0,01	0,01		0,02	0,03	0,022	0,013	0,013	0,017	0,019	0,015
Sulfate (mg/kg)	0,11			0,09	1,1	0,41	0,35	0,54	0,66	0,48	0,29	0,16	0,23	0,34		0,70	0,73	0,70	0,35	0,41	0,53	0,45	0,25
phenol index																							
pH	12,5	11,6	11,6	13,7	12,7	13,3	12,5	12,4	12,2	12,3	12,7	12,2	11,4	11,9	11,9	12,1	11,8	12,1	11,9	11,9	11,9	12,2	0,6
Conductivity (mS/m)	432	1094	1110	1006	722	660	609	489	371	380	312	350	104,5	183	168	219	189	163	135	135	136	427	324
L/S	0,0038	0,0063	0,0038	0,0047	0,054	0,021	0,018	0,027	0,033	0,024	0,015	0,008	0,011	0,017	0,038	0,035	0,036	0,035	0,018	0,021	0,026		
L/S (cumulative)	0,0038	0,0101	0,014	0,019	0,072	0,093	0,110	0,14	0,17	0,19	0,21	0,22	0,23	0,25	0,28	0,32	0,35	0,39	0,41	0,43	0,45		

Table S4
ICP-MS (Agilent 7900) operating parameters for the determination of element concentrations in aqueous leachates.

Parameter	Type/Value	Helium mode	HECM mode	No gas mode
Sample introduction				
Nebuliser	Micromist			
Spray chamber	Scott			
Skimmer and sampler	Ni			
Plasma conditions				
Forward power	1550 W			
Plasma gas flow	15.0 L min^{-1}			
Carrier gas flow		1.05 L min^{-1}	1.05 L min^{-1}	1.05 L min^{-1}
Makeup gas flow		0.10 L min^{-1}	0.10 L min^{-1}	0.10 L min^{-1}
He gas flow		4.5 mL min^{-1}	10 mL min^{-1}	
QP bias		-13.0 V	-93 V	-3.0 V
OctP bias		-18.0 V	-100 V	-8.0 V
Cell entrance		-40 V	-100 V	-30 V
Cell exit		-60 V	-150 V	-50 V
Deflect		1.6 V	-74 V	11.4 V
Plate bias		-60 V	-150 V	-35 V
Sample uptake rate	0.3 mL min^{-1}			
Data acquisition parameters				
Isotopes monitored		^{52}Cr, ^{55}Mn,^{56}Fe, ^{60}Ni, ^{63}Cu, ^{66}Zn, ^{75}As, ^{95}Mo,	^{78}Se	^{111}Cd, ^{123}Sb, ^{137}Ba, ^{201}Hg, ^{208}Pb
Isotopes of internal standards		^{72}Ge, ^{89}Y, ^{103}Rh, ^{115}In	^{72}Ge, ^{89}Y, ^{103}Rh, ^{115}In	^{72}Ge, ^{89}Y, ^{103}Rh, ^{115}In

Quality of analytical data and limits of detection

The quality of the analytical measurements of the PHSs in aqueous leachates and the samples of percolated water from the lysimeters by ICP-MS was checked with an analysis of the standard reference material SPS-SW1 (Reference material for measurements of elements in surface waters) obtained from Spectrapure Standards (Oslo, Norway) and measurements of Cl^-, F^- and SO_4^{2-} by spectrophotometry, by analysing reference material Anions – While Volume, Merck KGaA (Darmstadt, Germany). The results are provided in Tables 5S–6S.

To check the quality of the determination of total element concentrations in the composite sample by ICP-MS after microwave-assisted digestion, certified reference material CRM 320R Trace Elements in River Sediment (Community Bureau of Reference, Geel, Belgium) was analysed. The results provided in Tables 7S.

The results provided in Tables 5S–7S indicate that the determined concentrations of the elements showed a good correlation with the reported certified values (the agreement between the results was better than ± 5%), thereby confirming the accuracy of the analytical procedures applied. The expanded uncertainty for the ICP-MS determinations was better than ± 3% (k = 2) and for spectrophotometry it was better than ± 5% (k = 2).

The limits of detection (LODs) for the determination of elements by ICP-MS and anions by spectrophotometry in aqueous leachates and samples of percolated water from lysimeters as well as the total elemental concentrations by ICP-MS in the composite samples are provided in Table 8S.

Table S5

Certified and determined concentrations of PTEs in the standard reference material SPS-SW1 (Reference material for measurements of elements in surface waters) determined by ICP-MS. The results represent the mean concentration obtained from four parallel samples.

Parameter	Certified value (µg/L)	Determined (µg/L)
As	10.0±0.1	9.9±0.3
Ba	50±1	49±1
Cd	0.50±0.01	0.49±0.01
Total Cr	2.00±0.02	1.99±0.06
Cu	20±1	19.6±0.6
Hg	/	/
Mo	10.0±0.1	10.1±0.6
Ni	10.0±0.1	10.0±0.1
Pb	5.0±0.1	4.8±0.1
Sb	/	/
Se	2.00±0.02	2.02±0.06
Zn	20[b]	19.1±0.6

[b]informative value/not certified

Table S6

Concentrations of chlorides, fluorides and sulphates in the standard reference material Anions – While Volume obtained from Merck KGaA (Darmstadt, Germany) determined by spectrophotometry. The results represent the mean concentration obtained from four parallel samples.

Parameter	Certified (mg/L)	Determined (mg/L)
chlorides	95.0 ± 9.50	93.5 ± 5.0
fluorides	1.17 ± 0.117	1.10 ± 0.06
sulphates	44.3 ± 4.43	42.5 ± 2.0

Table S7

Concentrations of elements in certified reference material CRM 320R (Trace Elements in River Sediment) determined by ICP-MS after microwave-assisted digestion. The results represent the mean concentration from three parallel samples.

Parameter	Certified (mg/kg)	Determined (mg/kg)
As	21.7±2.0	20.4±0.6
Ba	/	/
Cd	2.64±0.18	2.52±0.07
Cr	59±4	62.7±1.3
Cu	46.3±2.9	44.2 ±0.9
Hg	/	/
Mo	/	/
Ni	27.1±2.2	26.6±0.6
Pb	85±5	81±3
Sb	/	/
Se	/	/
Zn	319±20	311±7

Table S8

LODs for the determination of element concentrations aqueous leachates and lysimetric waters by ICP-MS and anions by spectrophotometry, and determination of the total element concentrations in composite samples by ICP-MS.

Parameter	Aqueous leachates, lysimetric water samples (mg/kg)	Composite samples (mg/kg)
As	0.001	1.5
Ba	0.02	30
Cd	0.002	3
Total Cr	0.002	3
Cu	0.001	1.5
Hg	0.001	1.5
Mo	0.002	3
Ni	0.002	3
Pb	0.005	7.5
Sb	0.001	1.5
Se	0.003	4.5
Zn	0.005	1.5
Cl^-	2	/
F^-	1	/
SO_4^{2-}	10	/

Table S9

Total concentrations of PTEs in raw materials after microwave-assisted digestion determined by ICP-MS. The expanded uncertainty of the analytical procedure was better than ± 3% (k = 2).

Parameter (mg/kg)	Paper-mill sludge	Foundry sand	Paper ash	Coal ash	Solid-waste incineration bottom ash	Foundry slag	Digestate	Mine waste
As	0.39	5.8	1.3	3.0	11	8.37	17	4.1
Ba	17	268	65	332	2708	246	600	157
Cd	0.31	0.45	0.14	0.50	44	1.5	1.3	0.27
Cr	28	70	55	2	600	234	350	42
Cu	7.2	31	88	352	1500	35	600	10
Hg	0.31	1.1	0.2	0.2	12	1.2	/	0.6
Mo	2.7	4.0	3.4	3.0	160	7.2	19	7.9
Ni	6.4	23	9.4	12	113	32	130	15
Pb	12	32	17	76	1115	47	390	17
Sb	0.20	2.6	0.63	1.4	120	4.9	9.5	0.71
Se	20	9.3	3.9	4.0	8.0	8.0	1.3	18
Zn	20	3680	48	100	14734	2646	680	47

Table S10

Total concentrations of PTEs in composites after microwave-assisted digestion determined by ICP-MS. The results represent the mean concentration obtained from three parallel samples.

Element	Composite 1 (mg/kg)	Composite 2 (mg/kg)	Composite 3 (mg/kg)
As	10.8	3.90	3.10
Ba	911	431	138
Cd	2.03	0.5	0.10
Cr	150	125	76.2
Cu	171	334	20.4
Hg	0.69	0.22	0.02
Mo	18.8	5.63	3.62
Ni	85.3	36.7	20.3
Pb	123	356	15.4
Sb	6.44	10	0.88
Se	1.95	0.906	0.823
Zn	1140	313	510

Table S11
Concentrations of PTEs and anions in aqueous leachates from raw materials applying the SIST EN 1744-3:2002 leaching test. Limits for inertness, set by the current Slovenian legislation, are also given. Values highlighted with red exceed the threshold values for inertness. pH and electrical conductivity of aqueous leachates are also given. The expanded uncertainty of analytical procedure was better than ± 3% (k = 2).

Sample/ Parameter	Paper-mill sludge	Foundry sand	Paper ash	Digestate	Solid-waste incineration bottom ash	Foundry slag	Coal ash	Mine waste	Limit value*
As (mg/kg)	0.07	0.17	0.004	0.11	0.007	0.06	0.003	0.0007	0.1
Ba (mg/kg)	0.4	<0.02	63	2	1.6	0.11	48	0.0073	5
Cd (mg/kg)	<0.002	<0.002	<0.002	0.014	<0.002	<0.002	<0.002	<0.002	0.025
Cr (mg/kg)	0.006	<0.002	0.032	0.26	7.4	0.013	0.16	0.0022	0.5
Cu (mg/kg)	0.18	0.026	0.79	8.2	<0.001	0.012	0.003	0.0006	0.5
Hg (mg/kg)	<0.001	<0.001	0.003	0.0099	0.005	0.002	0.001	<0.0001	0.005
Mo (mg/kg)	0.15	0.16	0.77	1.2	8.3	0.013	0.17	0.0065	0.5
Ni (mg/kg)	0.11	0.015	0.002	1.3	0.002	0.004	<0.002	0.0010	0.4
Pb (mg/kg)	0.005	<0.005	0.051	1.7	<0.005	<0.005	0.054	<0.0005	0.5
Sb (mg/kg)	0.014	0.02	0.002	5	1.1	0.073	0.077	0.0001	0.3
Se (mg/kg)	0.004	0.06	0.003	0.017	0.15	0.026	0.031	0.0004	0.6
Zn (mg/kg)	0.18	0.03	0.043	10	<0.005	<0.005	0.23	0.0016	2
Cl^- (mg/kg)	89	116	59.8	3440	5830	473	438	27	800
F^- (mg/kg)	19	22	7.44	17.2	26.8	5.39	5.69	0.44	10
SO_4^{2-} (mg/kg)	290	580	<10	5860	1000	2000	<10	9	1000
Phenolic index (mg/kg)	0.25	<0.05	3.6	30	<0.20	<0.05	3.6	<0.05	0.1
pH	7.3	10.0	12.5	9.2	11.1	9.4	12.3	8.6	/
Conductivity (mS/m)	62	55	988	293	522	56	1164	16	/

* Decree on waste (Official Gazette of the Republic of Slovenia, nos. 37/15, 69/15 and 129/20)

Table S12

Concentrations of PTEs and anions in aqueous leachates from three uncompacted and compacted Composites 1, 2 and 3, applying SIST EN 1744-3:2002 leaching test. Results represent average concentrations of elements and anions obtained from three analysed samples for each composite. Limits for inertness, set by the current Slovenian legislation, are also provided. Values highlighted with red exceed the threshold values for inertness. pH and electrical conductivity of aqueous leachates are also given. The expanded uncertainty of analytical procedure was better than ± 3% (k = 2).

Sample/ Parameter	**Composite 1**		**Composite 2**		**Composite 3**		Limit value*
	Uncompacted	Compacted	Uncompacted	Compacted	Uncompacted	Compacted	
As (mg/kg)	0.033	0.028	0.072	0.039	0.0023	<0.001	0.1
Ba (mg/kg)	0.337	0.32	2.74	1.52	39.3	2.15	5
Cd (mg/kg)	<0.002	<0.002	<0.002	<0.002	<0.002	<0.002	0.025
Cr (mg/kg)	0.029	0.01	0.062	0.02	0.095	0.017	0.5
Cu (mg/kg)	0.048	0.03	2.85	4.8	0.0043	0.0017	0.5
Hg (mg/kg)	<0.001	<0.001	0.0017	0.0017	0.0023	<0.001	0.005
Mo (mg/kg)	0.0667	0.062		0.65	0.293	0.023	0.5
Ni (mg/kg)	0.023	0.013	0.49	0.58	<0.002	<0.002	0.4
Pb (mg/kg)	<0.005	<0.005	0.01	<0.005	0.053	<0.005	0.5
Sb (mg/kg)	0.0080	0.004	0.061	0.004	0.0023	0.0037	0.3
Se (mg/kg)	0.0050	0.0057	0.0083	0.079	0.015	0.0047	0.6
Zn (mg/kg)	0.067	0.058	0.125	0.0057	0.0650	0.0060	2
Cl^- (mg/kg)	274	323	1210	267	568	51.8	800
F^- (mg/kg)	5.3	7.1	10.6	8.7	6	0.7	10
SO_4^{2-} (mg/kg)	563	543	2667	273	137	137	1000
Phenolic index (mg/kg)	0.054	<0.01	7.9	0.42	<0.01	<0.01	0.1
pH	8.0	8.3	10.8	11.6	12.4	11.6	/
Conductivity (mS/m)	292	193	562	681	1158	495	/

* Decree on waste (Official Gazette of the Republic of Slovenia, nos. 37/15, 69/15 and 129/20)
