# Supplementary figures and images for: Environmental Acceptability of Geotechnical Composites from Recycled Materials: Comparative Study of Laboratory and Field Investigations
Source: Int J Environ Res Public Health. 2023 Jan 21;20(3):2014. doi: 10.3390/ijerph20032014 (PMC9915455; doi:10.3390/ijerph20032014)

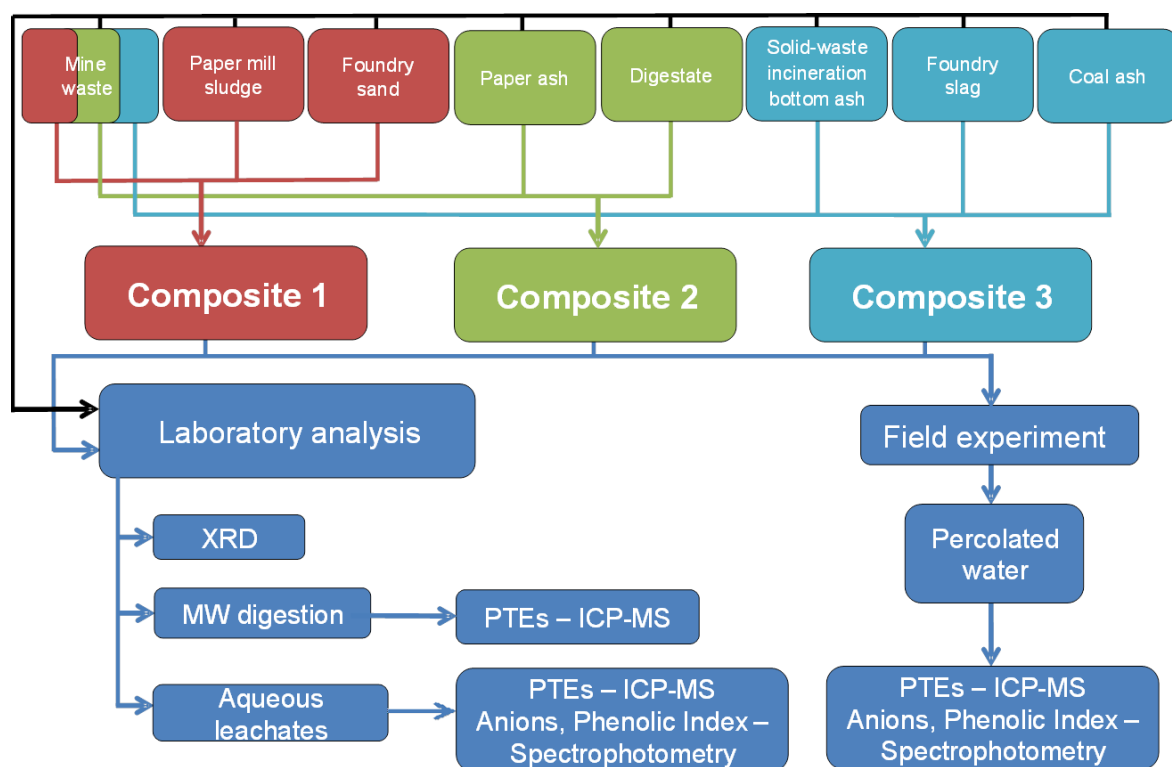

**Figure S1** A flow chart of the experimental set-up.

Supplement: Supplementary file 1 [file ijerph-20-02014-s001.zip › Figure 1S.pdf]
